# The within-host evolution of antimicrobial resistance in *Mycobacterium tuberculosis*

**DOI:** 10.1093/femsre/fuaa071

**Published:** 2020-12-15

**Authors:** Rhastin A D Castro, Sonia Borrell, Sebastien Gagneux

**Affiliations:** Swiss Tropical and Public Health Institute, Socinstrasse 57, 4051 Basel, Basel, Switzerland; University of Basel, Petersplatz 1, 4001 Basel, Basel, Switzerland; Swiss Tropical and Public Health Institute, Socinstrasse 57, 4051 Basel, Basel, Switzerland; University of Basel, Petersplatz 1, 4001 Basel, Basel, Switzerland; Swiss Tropical and Public Health Institute, Socinstrasse 57, 4051 Basel, Basel, Switzerland; University of Basel, Petersplatz 1, 4001 Basel, Basel, Switzerland

**Keywords:** *Mycobacterium tuberculosis*, evolution, within-host, antimicrobial resistance, virulence, genetic diversity, population dynamics

## Abstract

Tuberculosis (TB) has been responsible for the greatest number of human deaths due to an infectious disease in general, and due to antimicrobial resistance (AMR) in particular. The etiological agents of human TB are a closely-related group of human-adapted bacteria that belong to the *Mycobacterium tuberculosis* complex (MTBC). Understanding how MTBC populations evolve within-host may allow for improved TB treatment and control strategies. In this review, we highlight recent works that have shed light on how AMR evolves in MTBC populations within individual patients. We discuss the role of heteroresistance in AMR evolution, and review the bacterial, patient and environmental factors that likely modulate the magnitude of heteroresistance within-host. We further highlight recent works on the dynamics of MTBC genetic diversity within-host, and discuss how spatial substructures in patients’ lungs, spatiotemporal heterogeneity in antimicrobial concentrations and phenotypic drug tolerance likely modulates the dynamics of MTBC genetic diversity in patients during treatment. We note the general characteristics that are shared between how the MTBC and other bacterial pathogens evolve in humans, and highlight the characteristics unique to the MTBC.

## Teaser

Heteroresistance is an important stepping-stone to how an initially monoclonal and drug-susceptible population of *Mycobacterium tuberculosis* becomes fully resistant to a given antimicrobial during the course of an infection, and this review discusses how bacterial mutation rates, bacterial population size, the number of mutations that can confer antimicrobial resistance (i.e. AMR target size) and the fitness of AMR mutations all modulate the magnitude of heteroresistance.

## INTRODUCTION

For millennia, tuberculosis (TB) has been a scourge on humanity (Brites and Gagneux 2015). Today, TB remains a global burden on human health, being the leading cause of death due to an infectious disease in humans (Paulson 2013; WHO 2020). In 2019, there were approximately 10 million incident TB cases, with an estimated 1.2 million deaths due to TB alone and around 208 000 additional deaths due to TB–HIV co-infections (WHO 2020). TB in humans is generally caused by bacterial species that belong to the *Mycobacterium tuberculosis* complex (MTBC; Gagneux 2018). Although the MTBC genetic diversity is small compared to other bacteria, the global human-adapted MTBC populations can be currently grouped into nine phylogenetic lineages (Comas *et al*. 2010; Gagneux 2018; Coscolla *et al*. 2020; Ngabonziza *et al*. 2020a). These lineages differ in their phylogeographic distributions and phenotypic characteristics, which can modulate multiple aspects of virulence and antimicrobial resistance (AMR) evolution (Gagneux 2018; Coscolla *et al*. 2020; Peters *et al*. 2020; Ngabonziza *et al*. 2020a).

AMR in TB is of particular importance as it represents the single largest cause of mortality due to AMR, accounting for approximately 200 000 out of the nearly 700 000 AMR-related deaths in 2014 (O'Neill 2014). In general, AMR is an emerging global crisis as it increases treatment failures, treatment duration, treatment costs and the likelihood of adverse side effects from treatment (MacGowan 2008; Laxminarayan *et al*. 2013; Kibret *et al*. 2017; Zhang *et al*. 2018). AMR therefore imposes a severe economic and societal impact (O'Neill 2014; Roope *et al*. 2019). To treat TB infections, current first-line treatment against drug-susceptible TB uses a standardized, empirical dosing combination therapy of four drugs: isoniazid, rifampicin, pyrazinamide and ethambutol (WHO 2017). This first-line regimen has high efficiency in the clinic, with an approximately 85% treatment success (Farah *et al*. 2005; Bao, Du and Lu 2007; Gebrezgabiher *et al*. 2016; WHO 2020). Multidrug-resistance TB (MDR-TB), defined as an infection with an MTBC strain that is resistant to at least isoniazid and rifampicin, presents greater medical, economic and logistical challenges compared to drug-susceptible TB, as treatment is both longer and has lower success rates (Kibret *et al*. 2017; Zhang *et al*. 2018; Nunn *et al*. 2019; WHO 2020). Further difficulties arise when patients have extensively-drug resistant TB (XDR-TB), defined as cases of MDR-TB that have additional resistance to two of the current key second-line drugs: fluoroquinolones and injectable aminoglycosides (Leimane *et al*. 2010; Alene *et al*. 2017; WHO 2020). Understanding how AMR evolves in MTBC populations is therefore important to maintain our ability to treat patients and control TB.

Most studies on TB evolution have so far focused on between-host dynamics, most notably in efforts to trace transmission networks (Gardy *et al*. 2011; Walker *et al*. 2013; Nikolayevskyy *et al*. 2019) and to detect AMR mutations (Gygli *et al*. 2017; Cohen *et al*. 2019), with comparably few studies focusing on the within-host evolution of the MTBC. While multiple studies have elucidated how other bacterial pathogens evolve within patients (reviewed in Didelot *et al*. 2016), these have mainly focused on opportunistic infections, such as *Pseudomonas aeruginosa* (Winstanley, O'Brien and Brockhurst 2016; Clark, Guttman and Hwang 2018) and *Burkholderia dolosa* (Lieberman *et al*. 2014) in patients with cystic fibrosis. In contrast, the MTBC is a professional pathogen with no environmental reservoir, and exhibits extreme clonality compared to other bacterial pathogens (Achtman 2008; Brites and Gagneux 2015; Gagneux 2018). This likely leads to unique characteristics in how the MTBC evolves within patients.

In this review, we explore the recent advances that have shed light on the within-host evolution of the human-adapted MTBC, and discuss the population dynamics of AMR evolution during MTBC infections within-host. Specifically, we first highlight the unique characteristics of AMR in MTBC compared to other bacteria. We also highlight the phenomenon of ‘heteroresistance’ and its importance in the evolution of AMR in bacteria in general, and in the MTBC specifically. The bacterial populations’ capacity to generate and maintain genetic diversity modulates the magnitude of heteroresistance. Therefore, we also discuss how different biological factors modulate the generation and maintenance of genetic diversity in MTBC populations, and how they can modulate the magnitude of heteroresistance. Lastly, we review studies that focused on the MTBC genetic diversity dynamics in the context of within-host AMR evolution. We highlight the roles that spatiotemporal heterogeneity in antimicrobial concentration and bacterial density, as well as the potential role of phenotypic drug tolerance and bacterial persisters, might play on AMR evolution in the MTBC.

### AMR in the MTBC

Antimicrobials are substances that kill or stunt the growth of microbes by targeting essential or important biochemical mechanisms. AMR may be defined as when the pathogen population infecting a patient harbors a biochemical mechanism that allows them to survive and replicate when exposed to a concentration of antimicrobial to which they would otherwise be killed (Blair *et al*. 2015; Munita and Arias 2016). Thus, while the complex interaction between multiple behavioral, socioeconomic and health systems-related factors modulate the prevalence of AMR (Laxminarayan *et al*. 2013; Alvarez-Uria, Gandra and Laxminarayan 2016; Eldholm *et al*. 2016; O'Neill 2014), AMR is ultimately a biological process subject to evolutionary forces (zur Wiesch *et al*. 2011; Hughes and Andersson 2017).

Antimicrobial activity is dependent on the antimicrobial reaching and interacting with its target in the pathogen. Bacteria can exhibit AMR in two general ways: they are intrinsically resistant to the antimicrobial, or they can acquire new resistance determinants. AMR determinants in bacteria have been extensively reviewed in Blair *et al*. (2015) and Munita and Arias (2016), and recently in the MTBC by Gygli *et al*. (2017), and are therefore beyond the scope of this review. Here, we provide brief examples to show the AMR features unique to the MTBC.

Intrinsic resistance is when a given pathogen can survive exposure to an antimicrobial that is effective against other pathogens; this is due to inherent structural or biochemical mechanisms in the given pathogen that prevent antimicrobials from reaching or interacting with their target. For example, mycobacteria have a characteristic cell wall that is thicker and more hydrophobic than most other bacteria (Jankute *et al*. 2015; Dulberger, Rubin and Boutte 2020). Intracellular accumulation of antimicrobials in mycobacteria are therefore highly dependent on transit through porins embedded in the cell wall, but the transit of compounds was shown to be slower through the cell wall of mycobacteria compared to *Escherichia coli* and *P. aeruginosa* (Jarlier and Nikaido 1994; Liu *et al*. 1996). The mycobacterial cell wall has been shown to confer intrinsic resistance to many compounds, including antimicrobials, by acting as a considerable permeability barrier (Jarlier and Nikaido 1994; Gygli *et al*. 2017). The MTBC are also intrinsically resistant to the majority of β-lactam antibiotics, an important broad-spectrum antimicrobial class used against many other bacterial infections, as the MTBC genome encodes the extended spectrum beta-lactamase (ESBL) *blaC* (Hugonnet and Blanchard 2007; Tremblay, Fan and Blanchard 2010). Taken together, intrinsic resistance in the MTBC restricts the number of potential substances that can serve as antimicrobials compared to most other bacteria, complicating drug discovery and development efforts (Gygli *et al*. 2017). Notably, intrinsic resistance is particularly important in infections caused by nontuberculous mycobacteria (NTM; Luthra, Rominski and Sander 2018; Huh *et al*. 2019; Johansen, Herrmann and Kremer 2020). NTMs are a group of environmental mycobacteria related to the MTBC and the leprosy-causing bacteria *Mycobacterium leprae* and *Mycobacterium lepramatosis* (Fedrizzi *et al*. 2017). NTMs do not cause TB nor leprosy, but can cause a wide range of other infections, including pulmonary disease (particularly in individuals with pre-existing lung pathologies, such as cystic fibrosis patients) and skin and soft-tissue infections (Lee *et al*. 2015; Johansen, Herrmann and Kremer 2020). NTMs pose an emerging threat to public health due to an increasing number of infections reported (Lee *et al*. 2015; Johansen, Herrmann and Kremer 2020) while exhibiting intrinsic resistance to many antimicrobials, including antimicrobials that are active against the MTBC such as all first-line anti-TB drugs (Luthra, Rominski and Sander 2018; Huh *et al*. 2019; Johansen, Herrmann and Kremer 2020).

For infections caused by the MTBC, acquired resistance determinants pose the major challenge from a public health perspective, as previously effective treatment regimens are greatly reduced in their effectiveness or are rendered ineffective altogether (WHO 2020). In general, acquired resistance in bacteria can manifest through one or any combination of three mechanisms: (1) the modification of the antimicrobial target in the pathogen so that the antimicrobial cannot interact with or inhibit the target, (2) the reduction of the effective intracellular antimicrobial concentration by efflux or by upregulation of the antimicrobial target or (3) the inactivation of the antimicrobial itself (Blair *et al*. 2015; Munita and Arias 2016). These mechanisms may be acquired from one of two main sources: (a) the emergence of spontaneous mutations on the bacterial chromosome, or (b) the acquisition of genetic material harboring resistance genes from the environment, such as the horizontal-gene transfer (HGT) of mobile genetic elements (e.g. plasmids and transposons) between two different bacteria and the integration of the AMR genes into the recipient bacterial chromosome via recombination (Blair *et al*. 2015; Munita and Arias 2016). In the MTBC, HGT-based resistance has yet to be observed, as the MTBC lacks plasmid-based resistance (Gygli *et al*. 2017; Cohen *et al*. 2019), and the MTBC undergoes little recombination (Boritsch *et al*. 2016; Chiner-Oms *et al*. 2019). Indeed, the vast majority of clinically-relevant AMR determinants in the MTBC are derived from chromosomal mutations (Gygli *et al*. 2017; Cohen *et al*. 2019). These mutations can modify the structure of the antimicrobial target. For example, RNA polymerase modification confers rifampicin-resistance (McClure and Cech 1978; Campbell *et al*. 2001; Molodtsov *et al*. 2017), while modification of the type II topoisomerase DNA gyrase confers fluoroquinolone-resistance in the MTBC (Takiff *et al*. 1994; Piton *et al*. 2010). Notably, the MTBC does not encode topoisomerase IV, the other type II topoisomerase present in other bacteria and another target of fluoroquinolones (Cole *et al*. 1998). Chromosomal mutations may also modify the expression of the antimicrobial target. For instance, mutations leading to an overexpression of *inhA*, which encodes an NADH-dependent enoyl–acyl carrier protein, confers resistance to isoniazid and ethionamide (Vilchèze and Jacobs Jr 2014). Chromosomal mutations in the MTBC may also lead to AMR via upregulation of efflux pumps. For example, mutations in *Rv0678* have been shown to upregulate the expression of the MmpL5 efflux pump, which in turn confers resistance to the new drug bedaquiline and to clofazimine, an old drug originally used in leprosy and recently repurposed for MDR/XDR-TB (Andries *et al*. 2014; Hartkoorn, Uplekar and Cole 2014; Gygli *et al*. 2017). Recent work also showed that such *Rv0678* mutations have emerged repeatedly and even transmitted in southern Africa (Nimmo *et al*. 2020b). In the case of prodrugs like isoniazid and pyrazinamide, chromosomal mutations may confer AMR by decreasing the intracellular concentration of the active antimicrobial compound through reduced prodrug activation (Sreevatsan *et al*. 1997; Vilchèze and Jacobs Jr. 2014; Gygli *et al*. 2017).

When acquired resistance emerges from chromosomal mutations, as is the case in the MTBC, an AMR mutant may emerge from initially antimicrobial-susceptible population prior to antimicrobial exposure (Luria and Delbrück 1943; zur Wiesch *et al*. 2011; Hughes and Andersson 2017). Replication of this AMR mutant may lead to a stable or even increasing subpopulation of AMR mutants within a majority antimicrobial-susceptible population in the absence of antimicrobials (zur Wiesch *et al*. 2011; Hughes and Andersson 2017). The phenomenon of where a minority population with reduced antimicrobial susceptibility are present in a majority of susceptible population has been termed ‘heteroresistance’ (El-Halfawy and Valvano 2015). The presence and magnitude of heteroresistance is clinically important as it determines the pathogen population that can survive and proliferate in the presence of antimicrobial treatment, effectively modulating the likelihood of treatment failure due to AMR (Andersson, Nicoloff and Hjort 2019). In the next section, we discuss the phenomenon of heteroresistance in bacteria in general, and its particular importance in the evolution of AMR in the MTBC specifically.

### Heteroresistance in the MTBC

**Figure 1.**
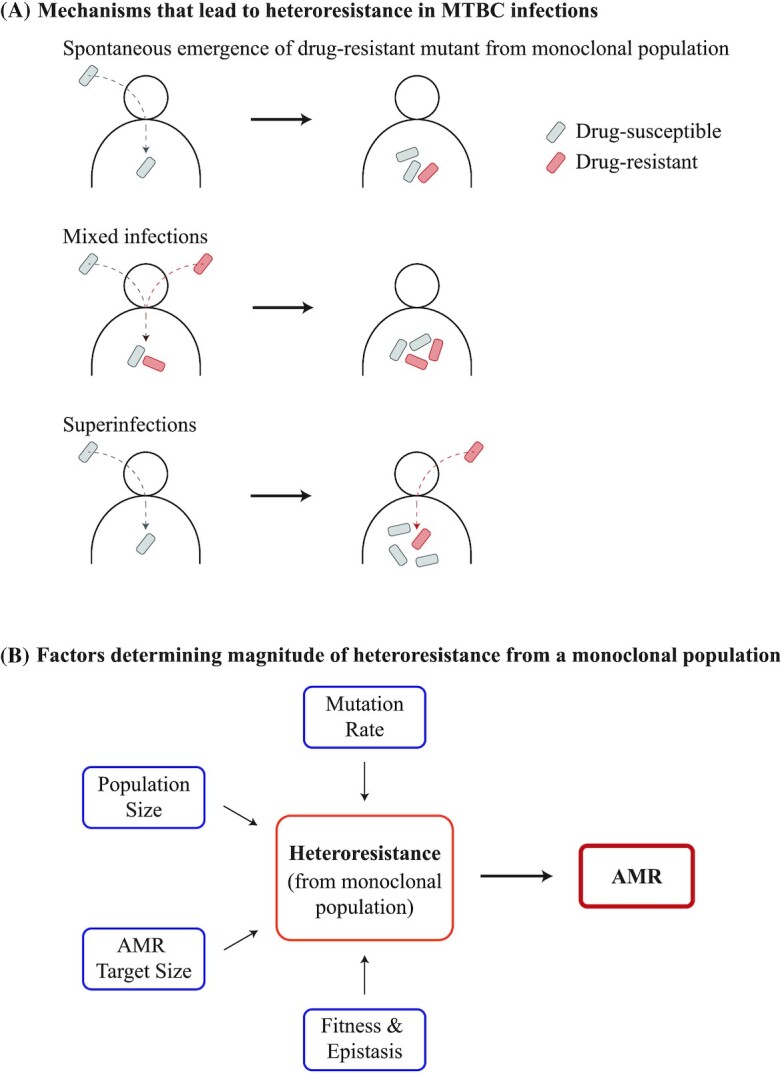
Heteroresistance in the MTBC. (A) Heteroresistance may manifest through three different mechanisms in MTBC infections. Firstly, an AMR mutant may spontaneously emerge from an initially monoclonal and drug-susceptible population. ‘Polyclonal’ infections may also lead to heteroresistance in the MTBC, of which two types are relevant: mixed infections and superinfections. Mixed infections are defined as when two different clones infect a given patient simultaneously. In contrast, superinfections are defined as an infection with one clone following a previous infection with a different clone that was not cleared over time. Polyclonal infections can lead to heteroresistance if one of the clones was an AMR mutant and the other was drug-susceptible. Of particular note, heteroresistance from gene duplications may be common in other bacteria, but have so far not been reported in the MTBC. (B) Bacterial mutation rates, bacterial population sizes, the number of mutations that can confer the AMR phenotype (i.e. AMR target sizes), and the fitness of AMR mutations can all determine the emergence and magnitude of heteroresistance from an initially drug-susceptible, monoclonal MTBC population, which in turn modulates the prevalence of AMR.

In clinical settings, heteroresistance in bacterial populations, including in the MTBC, can occur through multiple mechanisms. The first mechanism is the spontaneous emergence of AMR mutations from an initially drug-susceptible and monoclonal population (also known as ‘*de novo* resistance’; Fig. 1A). Heteroresistance can also occur through polyclonal infections. In the MTBC, two mechanisms can lead to heteroresistance from polyclonal infections: mixed infections and superinfections. Mixed infections occur when two or more different MTBC strains infect the same patient at the same time. Superinfections occur when a patient is infected by one MTBC strain, no bacterial clearance is achieved after some time and then the same patient is infected by an additional MTBC strain or multiple strains. The frequency of mixed infections occurring relative to the frequency superinfections occurring is currently not known. Nevertheless, in polyclonal infections caused by either mixed infections or superinfections, heteroresistance can occur if one infecting MTBC strain is drug-susceptible, and the other drug-resistant. Note that superinfections differ from reinfections, which refers to a patient infected by one MTBC strain that is then cleared prior to the patient being infected by another strain. Even though multiple MTBC genotypes might be observed in the same patient over time, formal heteroresistance cannot occur through reinfections alone as only a single strain is present at any one time. Notably, heteroresistance from polyclonal infections is ultimately the by-product of an AMR mutant initially emerging from drug-susceptible MTBC population in a given TB patient, and the AMR mutant becomes part of a polyclonal infection following a transmission event. Therefore, from an evolutionary standpoint, heteroresistance from polyclonal infections in MTBC is simply the result of a previous heteroresistance gained from the spontaneous emergence of AMR mutations. We will therefore focus on the latter scenario.

It is worth noting that gene amplification events may also lead to heteroresistance in an initially drug-susceptible and monoclonal population (Nicoloff *et al*. 2019). This occurs if the region amplified contains a gene that leads to AMR in a gene dosage manner, such as the duplication of a gene encoding an efflux pump (Nicoloff *et al*. 2019). Recent work showed that the tandem amplification of AMR genes may occur and be lost frequently in bacterial cells within an initially drug-susceptible population, leading to transient heteroresistance (Nicoloff *et al*. 2019). This was shown to be a major source of heteroresistance in four different Gram-negative species: *E. coli*, *Klebsiella pneumonia*, *Salmonella enterica* serovar Typhimurium and *Acinetobacter baumannii* (Nicoloff *et al*. 2019). However, heteroresistance from gene amplifications have yet to be observed in the MTBC. Hypothetically, transient amplification and increased expression of *inhA* could result in heteroresistance to isoniazid and ethionamide (Vilchèze and Jacobs Jr 2014). Similarly, transient amplification and increased expression of the MmpL5 efflux pump could result in heteroresistance to bedaquiline and clofazimine (Andries *et al*. 2014; Hartkoorn, Uplekar and Cole 2014).

Heteroresistance from the spontaneous emergence of AMR mutants in the MTBC has been documented since the discovery of antimicrobials, with experiments in 1947 showing large *in vitro* cultures of H37Rv exhibiting heteroresistance to streptomycin (Vennesland, Ebert and Bloch 1947). While heteroresistance in sputum samples were previously identified using PCR amplification of AMR genes followed by restriction fragment length polymorphism of the PCR products (Rinder, Mieskes and Löscher 2001), DNA sequencing technologies, including next generation sequencing (NGS) and whole genome sequencing in particular (WGS), have greatly enhanced our ability to detect heteroresistance (Box 1). Indeed, DNA sequencing have identified heteroresistance in clinical samples for practically every anti-tubercular drug, including streptomycin (Mariam *et al*. 2011), isoniazid (Sun *et al*. 2012; Operario *et al*. 2017; Metcalfe *et al*. 2017a), rifampicin (Sun *et al*. 2012; Operario *et al*. 2017; Metcalfe *et al*. 2017a), pyrazinamide (Operario *et al*. 2017), ethambutol (Operario *et al*. 2017; Nimmo *et al*. 2019), fluoroquinolones (Eilertson *et al*. 2014; Operario *et al*. 2017; Metcalfe *et al*. 2017a; Rigouts *et al*. 2019), injectable aminoglycosides (Operario *et al*. 2017; Metcalfe *et al*. 2017a), bedaquiline (de Vos *et al*. 2019) and delamanid (Bloemberg *et al*. 2015). However, even WGS does not detect all genetic variants that exist at very low frequencies (Comas 2017; Meehan *et al*. 2019). Thus, our ability is limited in fully elucidating the population dynamics of heteroresistance within patients. As with any evolutionary process, the rate at which genetic diversity is produced and maintained in a given pathogen population and environment modulates AMR emergence. Thus, in the next section, we use evolutionary principles to hypothesize how different biological factors may be relevant in determining the rate of AMR emergence and, consequently, the magnitude of heteroresistance following infection with an initially drug-susceptible MTBC strain.

Box 1. **Methodological considerations in using DNA sequencing technologies to study within-host MTBC evolution**

The advent of DNA sequencing technologies, including NGS and WGS in particular, has revolutionized our ability to study the genetic diversity in the MTBC. Methodological aspects, advances and limitations in using NGS to study the MTBC were recently reviewed in (Comas 2017) and (Meehan *et al*. 2019). Here, we highlight six relevant considerations in using DNA sequencing to measure the MTBC genetic diversity and study AMR evolution within-host.

Firstly, sputum samples are the current gold standard to study MTBC evolution. However, the MTBC genetic diversity in individual sputum samples are likely not representative of the overall diversity present in the lungs, as different granulomas within the same patient may contribute differently to the bacterial genetic diversity in patient sputa (Shamputa *et al*. 2006; Cadena, Fortune and Flynn 2017). Indeed, sputum samples from the same patient isolated on the same day have shown differences in MTBC genetic diversity (Pérez-Lago *et al*. 2014; Trauner *et al*. 2017). Thus, multiple sputum samples may be required to capture as much of the bacterial genetic diversity present within patients’ lungs.

A total of two general methods are used to measure the genetic diversity present in samples: (1) targeted or amplicon sequencing, which sequences only a selected group of loci (such as AMR genes) and (2) WGS, which sequences the entire genome. Amplicon sequencing does not require regrowing of the bacteria from sputum samples, which when combined with the lower complexity of sequencing data, provides a faster, cheaper, easier to analyze and more scalable sequencing technique than WGS (Colman et al. 2016, 2019; Jones and Good 2016). However, amplicon sequencing generally requires *a priori* knowledge of the loci of interest, such as AMR genes (Jones and Good 2016). In contrast, WGS provides unparalleled resolution in detecting the entire genetic diversity present in a given sample (Goodwin, McPherson and McCombie 2016; Comas 2017; Meehan *et al*. 2019). Compared to amplicon sequencing, WGS allows for a more thorough study of the evolution of populations (as reviewed in this work, notably in the section ‘Dynamics of Genetic Diversity in Presence of Antimicrobials’), as well as for the initial identification of AMR genes (Gygli *et al*. 2017; Cohen *et al*. 2019) and loci where mutations may potentiate the acquisition of AMR (Hicks *et al*. 2018; Bellerose *et al*. 2019; Safi *et al*. 2019).

Regrowing bacteria from sputum either in liquid or on solid media prior to DNA extraction for WGS may also modulate the bacterial genetic diversity observed. Regrowth allows for greater MTBC DNA yields, but may result in the loss of genetic diversity through (1) genetic drift, (2) outcompeting of low-fitness variants or (3) adaptation to *in vitro* conditions (Martín *et al*. 2010; Hanekom *et al*. 2013; Metcalfe *et al*. 2017b). Regrowth therefore leads to lower likelihoods of identifying heteroresistance (Metcalfe *et al*. 2017b). In contrast, directly sequencing from sputum increased the likelihood of identifying minor genetic variants (Nimmo *et al*. 2019; Shockey, Dabney and Pepperell 2019), increased the ability to detect heteroresistance (Metcalfe *et al*. 2017b) and decreased the time required to determine, to which drugs a given MTBC sample shows resistance (Doyle *et al*. 2018). However, direct sputum sequencing is susceptible to contamination and low MTBC DNA yields (Votintseva *et al*. 2017; Doyle *et al*. 2018). Recently, Soundararajan *et al*. (2020) sequenced directly from sputum and used a DNA-enrichment step during library preparation to provide sequencing reads that mapped to 85% of the MTBC genome with a 300-fold average coverage. Such a technique may prove useful in sequencing MTBC bacilli directly from sputum.

How samples are sequenced may also determine the MTBC genetic diversity observed in patient samples. A total of two general methods for performing WGS on samples have been performed: (1) pooled or metagenomic sequencing and (2) single-colony sequencing. Pooled sequencing may be done using the entire population present within a liquid culture, or from scraping all colonies together from solid medium (Lieberman *et al*. 2014; Meehan *et al*. 2019). In either case, DNA from the entire sample is pooled and sequenced together, allowing for higher throughput, lower costs and the potential to identify all genetic diversity within the sample. However, pooled sequencing is limited by its inability to differentiate between individual haplotypes (i.e. differentiating given bacterial clones within the population). In contrast to pooled sequencing, single colonies may be isolated and sequenced separately. Single colony sequencing allows for a more robust differentiation of haplotypes, the identification of rare haplotypes that may be missed using pooled sequencing and a more thorough study of their population dynamics (Lieberman *et al*. 2014; Black *et al*. 2015; Liu *et al*. 2020b). However, single-colony sequencing is inherently more challenging logistically, as DNA extraction must be performed for many single colonies (Lieberman *et al*. 2014; Black *et al*. 2015; Liu *et al*. 2020b).

Depth of sequencing coverage is another important factor, as higher sequencing depths allow for greater resolution of the different MTBC genetic variants present in the sample. Current multiplexing sequencing methods normally provide MTBC sequencing depths that range between 50- and 80-fold coverage to allow for higher throughput and lower costs (Comas 2017; Meehan *et al*. 2019). In contrast, deep sequencing provide sequencing depths of >300-fold and allows for higher probabilities of capturing minor genetic variants and measuring their true proportions in the sample population (Liu *et al*. 2015; Trauner *et al*. 2017; Worby, Lipsitch and Hanage 2017; Lee *et al*. 2020). However, deep sequencing come at a higher cost and lower throughput than ‘normal-depth’ sequencing (Comas 2017; Logsdon, Vollger and Eichler 2020), and must also account for increased false-positive rates from higher chances of detecting PCR and sequencing errors (Nimmo *et al*. 2020a).

Lastly, the length of sequencing reads may modulate the MTBC genetic diversity observed in patients. Short-read sequencing generally provides read lengths ranging from 150 to 300 bp, and has been the gold standard as it provided higher throughput and lower costs than long-read sequencing (Goodwin, McPherson and McCombie 2016; Comas 2017). However, short-read sequencing have difficulties resolving the sequence in regions with long and repetitive sequences (such as the PE/PPE gene families), and resolving the lengths of long insertions and deletions (Goodwin, McPherson and McCombie 2016; Comas 2017). In contrast, long-read sequencing can provide reads that exceed multiple megabases in length, allowing for the determination of sequences in regions with long and repetitive sequences, as well as large insertions and deletions, that short-read sequencing cannot resolve (Bainomugisa *et al*. 2018; Dixit *et al*. 2019; Logsdon, Vollger and Eichler 2020). Recent advances and cost reductions may make long-read sequencing more accessible (Logsdon, Vollger and Eichler 2020).

### Bacterial generation and maintenance of genetic diversity

Here, we dedicate each subsection to four relevant bacterial factors that determine the emergence and maintenance of genetic diversity and, consequently, modulate the prevalence and magnitude of heteroresistance: the bacterial mutation rate, the effective bacterial population size, the bacterial mutational target size for AMR and the fitness of AMR mutations (Fig. 1B). We also discuss how host factors may modulate each factor.

### Role of mutation rates

The rate at which bacterial genetic diversity is produced can modulate the emergence of AMR mutations (zur Wiesch *et al*. 2011; Hughes and Andersson 2017). Genetic diversity in bacterial populations can be generated through DNA replication errors or DNA repair mechanism-induced mutagenesis, which together make up the DNA mutation rate (Denamur and Matic 2006; Singh 2017; Warner *et al*. 2017). Increased bacterial mutation rates have been positively associated with increased AMR prevalence *in vitro* and in natural populations of multiple bacterial species (Oliver *et al*. 2000; Chopra, O'Neill and Miller 2003; Örlén and Hughes 2006; Oliver and Mena 2010; Couce, Rodríguez-Rojas and Blázquez 2015). However, studies testing the role of mutation rates in determining the prevalence of AMR in MTBC have provided contradicting results; these studies have mainly focused on Lineage 2 (L2) ‘Beijing’ strains, as L2 Beijing strains have been repeatedly associated with multidrug resistance (Borrell and Gagneux 2009; Casali *et al*. 2014; Merker *et al*. 2015; Eldholm *et al*. 2016; Wollenberg *et al*. 2017). An initial genetic study by Ebrahimi-Rad *et al*. (2003) hypothesized that this association may be due to mutations in DNA repair enzymes that lead to hypermutator phenotypes in L2 Beijing. The authors highlighted four homologs of the *E. coli* DNA repair enzyme gene *mutT* present in the MTBC genome, with the *mutT2* and *mutT4* genes having the highest sequence match to their *E. coli* counterparts. More importantly, the authors found nonsynonymous mutations in *mutT2* and *mutT4*, as well as in the DNA repair enzyme gene *ogt*, which were specific to L2 Beijing strains, and thus in line with their hypothesis. However, while mutations in *mutT* do confer hypermutator phenotypes in *E. coli* (Denamur and Matic 2006; Oliver and Mena 2010; Wielgoss *et al*. 2013), this has not been confirmed in the MTBC. Indeed, a review by McGrath *et al*. (2014) highlighted functional work suggesting that mutations in the MTBC *mutT2* are unlikely to contribute to the same hypermutator phenotypes as when mutations are present in *E. coli mutT* (Moreland *et al*. 2009; Sang and Varshney 2013).

Recent works showed that mutations in the *nucS* gene, which encodes a putative endonuclease, conferred a hypermutator phenotype in *Mycobacterium smegmatis*, a species frequently used as a non-pathogenic model organism to study the MTBC (Castañeda-García et al. 2017, 2020), as well as in two other Actinobacteria: *Streptomyces coelicolor* (Castañeda-García *et al*. 2017) and the industrially important *Corynebacterium glutamicum* (Ishino *et al*. 2018; Takemoto *et al*. 2018). NucS is hypothesized to serve as the primary mismatch repair (MMR) system to detect and repair incorrectly matched DNA base pairs in Actinobacteria, which include the MTBC and *M. smegmatis*, as well as in many Archaea species (Castañeda-García *et al*. 2017; Ishino *et al*. 2018; Takemoto *et al*. 2018). This is because Actinobacteria and many Archaea species lack the canonical MutS-MutL-mediated MMR pathway used by most other bacteria (Castañeda-García *et al*. 2017; Ishino *et al*. 2018; Takemoto *et al*. 2018). However, whether mutations in *nucS* indeed confer a hypermutator phenotype in the MTBC, and whether this would lead to increased heteroresistance in the clinic, has yet to be directly tested. To date, only mutations in the PHP domain of *dnaE1* are confirmed to confer a hypermutator phenotype in the MTBC (Rock *et al*. 2015). These mutations were found in approximately 3% of tested MTBC clinical isolates, but did not appear to be specific to L2 Beijing strains. A given *dnaE1* mutation, DnaE1 Lys95Asn, found in a clinical MTBC strain increased its mutation rate by 3-fold, but whether or not this or any other naturally-occurring mutations in the PHP domain of *dnaE1* generally led to increased heteroresistance was unclear (Rock *et al*. 2015).

MTBC mutation rates as measured by fluctuation analysis have also provided contradicting results. While Ford *et al*. (2013) showed that L2 Beijing strains had higher rates of isoniazid-, rifampicin- and ethambutol-resistance acquisition compared to Lineage 4 (L4) strains, multiple other studies have provided contradicting results. An earlier study by Werngern and Hoffner, and a more recent study by Carey *et al*. (2018) have shown that L2 Beijing strains had the same frequency of rifampicin-resistance as other strains (Werngren and Hoffner 2003). Furthermore, while different MTBC strains can have between 10- and 100-fold difference in their frequencies of isoniazid- (Carey *et al*. 2018) or fluoroquinolone-resistance (Castro *et al*. 2020), MTBC strains with the highest AMR frequencies were not L2 Beijing. Recent work in *Mycobacterium leprae* showed that strains exhibiting particularly long branch lengths in phylogenetic trees may be indicative of a hypermutator phenotype (Benjak *et al*. 2018). However, such a phenomenon has yet to be observed for L2 strains specifically, or in the MTBC in general. Lastly, a recent study that performed a systematic study on the molecular clock of the MTBC using genomic sequences from 6285 strains suggested that while L2 strains did indeed have a higher molecular clock rate than L4 strains, L1 strains had clock rates that were comparable to L2 (Menardo *et al*. 2019). Thus, current experimental and phylogenomic evidence do not support the L2 Beijing hypermutator hypothesis.

Within a host environment, differences in bacterial physiology and metabolism may also modulate the frequency of mutations. Specifically, whether bacteria are actively replicating or under lower metabolic activity may influence the number of mutational events. It was previously assumed that actively replicating MTBC are associated with active TB disease, while MTBC under low metabolic activity are associated with latency (although little evidence supports this assumption; Lipworth *et al*. 2016; Behr, Edelstein and Ramakrishnan 2018). Using a macaque infection model, Ford *et al*. (2011) showed that the bacterial populations acquired the same number of mutations per day regardless of whether the macaques had active, latent, or reactivated TB disease. This work suggested that any effect of reduced replication during latent TB on lowering the number of mutations may be offset by larger number of mutations due to increased oxidative DNA damage in latent TB compared to active TB disease. Whether the increased oxidative DNA damage during latent TB was due to a stronger host immune response during latent TB or due to reduced bacterial DNA repair mechanism activity was unclear. Nevertheless, the authors suggested that this appreciable mutation rate during latency may predispose MTBC populations to becoming AMR with a similar likelihood as during active TB disease.

Whether MTBC populations causing latent TB infections in humans would also have appreciable rates of genetic diversity production is unclear. WGS of clinical MTBC samples have so far provided contradicting results, with some studies showing similar mutation rates in MTBC samples collected from latent TB as those in active TB disease (Lillebaek *et al*. 2016), while others showing that latent TB disease had lower mutation rates than active TB disease (Colangeli *et al*. 2014). Thus, more work is required to test for an association between MTBC mutation rates and TB disease state.

Oxidative DNA damage during active TB disease may itself modulate MTBC mutation rates. Recent work by Liu *et al*. (2020b) showed that some MTBC subpopulations can exhibit elevated mutation rates compared to other subpopulations within the same patient. The authors further suggest that these elevated mutation rates were likely due to reactive oxygen species (ROS)-induced mutagenesis, which was likely a consequence of the host immune response against the MTBC infection. However, whether or not ROS-induced mutagenesis translates to increased prevalence of heteroresistance in these MTBC subpopulations is still unclear.

Exposure to antibiotics may also modulate observed bacterial mutation rates. Fluoroquinolones are a notable example, as sub-lethal levels of fluoroquinolones have repeatedly been shown to lead to a dose-dependent increase in mutation rates in *E. coli* and in some strains of *Salmonella enterica* serovar Typhimurium (Ysern *et al*. 1990; Cirz *et al*. 2005; Kohanski, DePristo and Collins 2010; Pribis *et al*. 2019; Rodríguez-Rosado *et al*. 2019). Fluoroquinolones kill bacterial cells by binding to type II topoisomerases and generating double-stranded DNA breaks (DSBs) on the bacterial chromosome (Aldred, Kerns and Osheroff 2014). The increased levels of DSBs due to sub-lethal fluoroquinolone concentrations induces the SOS response, which in turn increases the expression of error-prone DNA polymerases, leading to the higher observed mutation rates (Ysern *et al*. 1990; Cirz *et al*. 2005; Rodríguez-Rosado *et al*. 2019). Notably, this mutagenic response is dependent on the production and downstream signalling of ROS (Kohanski, DePristo and Collins 2010; Pribis *et al*. 2019). Although sub-lethal fluoroquinolone exposure also increased mutation rates in laboratory strains of *P. aeruginosa*, clinical strains of *P. aeruginosa* showed little or no increase in mutation rates when exposed to the same sub-lethal fluoroquinolone concentration (Migliorini *et al*. 2019). The mutagenic effects of fluoroquinolones on the MTBC have yet to be tested. Sub-lethal concentrations of fluoroquinolones were shown to increase the frequency of AMR acquisition to multiple antibiotics in *Mycobacterium fortuitum* (Gillespie *et al*. 2005) and increased the expression of the SOS response and DNA repair genes in the laboratory MTBC strain H37Rv (O'Sullivan *et al*. 2008). However, the mutagenic effects of fluoroquinolones have yet to be tested on clinical strains of the MTBC.

Even if fluoroquinolones or other antimicrobials increased mutation rates, this may not always translate to higher genetic diversities. This is because the likelihood of observing new genetic variants in a given population is also dependent on the size of the population itself. We discuss the role of population size and dynamics in determining the genetic diversity in bacterial populations in the next subsection, using exposure to fluoroquinolones as the first example.

#### Role of population size

While population sizes does not modulate the rate of genetic diversity production *per se*, larger population sizes associate with increased genetic diversity simply due to a higher likelihood of genetic variants being present, as well as new ones emerging due to the larger number of replication events (Ellegren and Galtier 2016). A recent study by Frenoy and Bonhoeffer tested the ability of bacterial populations to harbor new genetic diversity when exposed to sub-lethal concentrations of bactericidal antibiotics, including fluoroquinolones, while taking into account the antibiotic's effect on population dynamics (Frenoy and Bonhoeffer 2018). Firstly, the authors showed that *E. coli* populations experienced appreciable rates of cell death under sub-lethal antibiotic concentrations, and that previous works may have overestimated mutation rates if death rates were not taken into account (Kohanski, DePristo and Collins 2010). Secondly, Frenoy and Bonhoeffer showed that even after controlling for the death rate, sub-lethal concentrations of a fluoroquinolone still induced higher mutation rates. This observation has been supported by recent work by Pribis *et al*. (2019). However, Frenoy and Bonhoeffer also showed that sub-lethal concentrations of fluoroquinolones may actually lead to lower genetic diversities, as fluoroquinolones caused a strong reduction in the bacterial population size. This in turn led to a rapid loss of new genetic variants, and reduced likelihoods of new genetic variants emerging. In the MTBC, fluoroquinolones also caused strong and rapid reductions in population size (Gosling *et al*. 2003; Nuermberger *et al*. 2004; Donald and Diacon 2008). Thus, while fluoroquinolones do have a mutagenic effect on bacteria, whether this translates to increased genetic diversity and, consequently, higher likelihoods of heteroresistance in the MTBC requires further investigation. Such studies should control for the antibiotic's effect on population dynamics.

Strain-dependent differences in MTBC population sizes within the host (i.e. bacterial load) may also lead to differences in the level of genetic diversity. Currently, no good estimates for the MTBC bacterial loads in human hosts have been proposed, as it is inherently difficult to accurately measure the bacterial population size in the lungs of TB patients. However, animal models have shown that different MTBC genotypes can differ in bacterial load inside host tissues (López *et al*. 2003; Dormans *et al*. 2004; Tsenova *et al*. 2005; Aguilar *et al*. 2010; Krishnan *et al*. 2011; Via *et al*. 2013). Such variation in bacterial loads may lead to differences in heteroresistance and, consequently, AMR prevalence. Indeed, increased lung bacterial loads may have led to the association between MDR-TB and L2 Beijing strains. Higher bacterial loads could hypothetically lead to faster rates of progression to active disease, higher likelihoods of transmission and consequently increased likelihood of being exposed to antimicrobials. However, lung bacterial loads in mouse models have shown contradicting evidence. While L2 Beijing strains could have higher lung bacterial loads than other non-Beijing strains (López *et al*. 2003), in a separate study, one of the L2 Beijing strains had the lowest (Dormans *et al*. 2004). Another study showed that L2 Beijing strains that had been transmitted between human patients in the clinic also had higher lung CFUs in mouse models compared to L2 Beijing strains that were classified as non-transmitters (Aguilar *et al*. 2010). In contrast, a similar study found the opposite phenomenon with L4 strains, where high-transmitting L4 strains instead had lower lung CFUs in mouse models than low-transmitting L4 strains (Verma *et al*. 2019). Taken together, these findings suggest important strain-dependent differences in bacterial load, and that different lineages may have different associations between bacterial load and transmissibility. Thus, more work is required to determine whether different MTBC strains or lineages require different bacterial loads to promote the onset of symptoms and transmission. Further, if and how MTBC strain- or lineage-dependent bacterial loads modulate the magnitude of heteroresistance *in vivo* is unclear.

#### Role of mutational target sizes for AMR

The mutational target size for AMR may also modulate the magnitude of heteroresistance. The AMR mutational target size may be defined as the total number of potential mutations available that can confer the AMR phenotype, with larger mutational target sizes leading to higher likelihoods of heteroresistance (Ford *et al*. 2013; Hughes and Andersson 2017). In the MTBC, differential AMR mutational target sizes may lead to the differences in the frequency of resistance between different drug classes, such as differences in the relative frequency of isoniazid- versus rifampicin-resistance (McGrath *et al*. 2014). Rifampicin binds to the β subunit of bacterial RNA polymerase, encoded by *rpoB*, and kills bacteria by preventing transcription through inhibition of RNA elongation (McClure and Cech 1978; Campbell *et al*. 2001; Molodtsov *et al*. 2017). Because RNA polymerase is an essential enzyme, the majority of rifampicin-resistance is conferred by nucleotide substitutions that occur in a specific 81-bp region of *rpoB* and still provide a functional RNA polymerase; this region has been termed the rifampicin-resistance-determining region (Telenti *et al*. 1993; Ramaswamy and Musser 1998). In contrast, isoniazid-resistance may be conferred through multiple mechanisms. Isoniazid is a prodrug that needs to be activated by the bacterial peroxidase-catalase encoded by *katG*, and the active compound prevents mycolic acid synthesis by inhibiting the bacterial enoyl–acyl-carrier-protein reductase encoded by *inhA* (Vilchèze and Jacobs Jr 2019). While many mutations have been observed to associate with isoniazid-resistance in the clinic, mutations in *katG* (including insertions, deletions or point mutations), mutations in *inhA* and point mutations in the promoter region of *inhA* have been the only mutations shown to definitively confer isoniazid-resistance (Ramaswamy and Musser 1998; Vilchèze and Jacobs Jr 2014; Gygli *et al*. 2017; Cohen *et al*. 2019). Still, the *in vitro* mutational target size for isoniazid-resistance has been shown to be much larger than rifampicin-resistance, which likely leads to the observation that isoniazid-resistance selected *in vitro* is consistently one- to two-orders of magnitude more frequent than rifampicin-resistance (David 1970; Bergval *et al*. 2009; Ford *et al*. 2013; McGrath *et al*. 2014). Further, despite drug-susceptible TB being treated with isoniazid and rifampicin simultaneously, isoniazid-resistance is significantly more prevalent than rifampicin-resistance in the clinic (Dean *et al*. 2020), and isoniazid-resistance is generally acquired prior to rifampicin-resistance (Manson *et al*. 2017). However, whether the *in vivo* mutational target size (i.e. the mutational target size during infection) for isoniazid-resistance is indeed larger than rifampicin-resistance has yet to be directly tested. Indeed, although KatG may be non-essential *in vitro* (Pym, Saint-Joanis and Cole 2002; Sassetti and Rubin 2003), mutations that abrogate its catalase-peroxidase activity may be so metabolically costly in the stressful environment inside macrophages that such mutations confer a high fitness cost *in vivo* (Bergval *et al*. 2009; Brossier *et al*. 2016); this could lead to a more restrictive AMR *in vivo* mutational target size for isoniazid-resistance compared to what is observed *in vitro*.

While the AMR mutational target size may lead to differences in the frequency of resistance between two different drug classes, there may also be MTBC genotype-dependent AMR mutational target sizes for the same drug. This may lead to lineage- or strain-dependent differences in heteroresistance and AMR prevalence for a given drug. Indeed, it has been shown that the *in vitro* mutational target size for rifampicin-resistance was larger in L2 Beijing strains compared L4 strains (Ford *et al*. 2013). Recent work has also shown strain-dependent AMR mutational target sizes and mutational profiles for fluoroquinolone-resistance in the MTBC *in vitro* (Castro *et al*. 2020). Further testing is required to determine whether AMR mutational target sizes *in vivo* are also dependent on the MTBC genotype, and whether MTBC genotype-dependent AMR mutational target sizes could lead to differences in the magnitude of heteroresistance and AMR in general *in vivo*.

#### Role of fitness and epistasis

**Figure 2.**
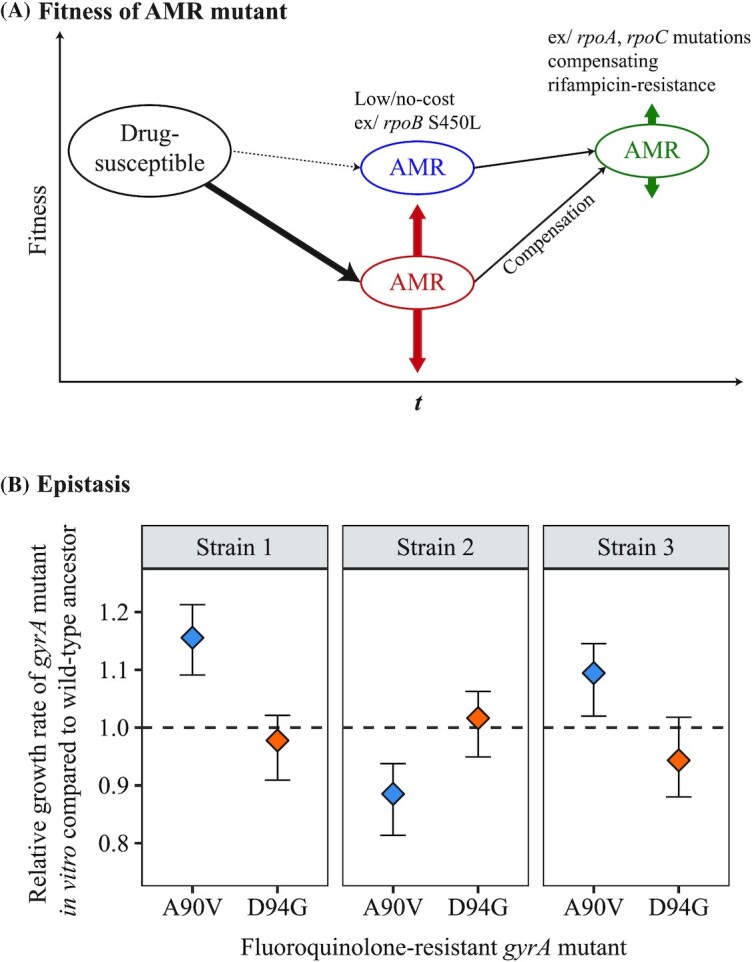
Fitness and Epistasis in AMR evolution. (A) The relative fitness of AMR mutants can modulate their relative frequency. Mutants with AMR mutations that confer little or no fitness cost are more likely to be maintained in the population, as low-cost AMR mutants can replicate at similar rates as their wild-type, drug-susceptible counterparts. However, most AMR mutations impart a fitness cost, with the magnitude of the fitness cost depending on the given mutation present and the genomic environment it is present in. Mutants with high-cost AMR mutations are less likely to be maintained in the population due to their lower replication rates. Further acquisition of secondary, compensatory mutations may alleviate fitness costs imparted by AMR mutations. (B) The fitness effects of AMR mutations may vary due to epistatic interactions with the MTBC genetic background. Here, *in vitro* growth rates were used as a measure of fitness, and the fitness of six different fluoroquinolone-resistant MTBC strains harboring either the fluoroquinolone-resistance-conferring *gyrA* A90V mutation or the *gyrA* D94G mutation were plotted relative to the fitness of their respective wild-type ancestor (dashed line = fitness of wild-type Strain 1, Strain 2 or Strain 3, respectively). The fitness effect of the *gyrA* A90V mutation depended on which MTBC strain they were present, while the fitness effect of the *gyrA* D94G mutation was similar in the three strain backgrounds tested (Fig. 2B adapted from Castro *et al*. 2020, with permission).

The fitness effect of AMR mutations may modulate the magnitude of heteroresistance. Because antimicrobials generally target essential and evolutionarily-conserved biomolecules or pathways, AMR mutations usually a confer fitness cost in antimicrobial-free environments (Andersson and Hughes 2010; Melnyk, Wong and Kassen 2015; Fig. 2A). However, in many bacterial species, fitness costs for AMR mutations have been found to vary greatly (Andersson and Hughes 2010; Melnyk, Wong and Kassen 2015; Vogwill and MacLean 2015; Leónidas Cardoso *et al*. 2020). Because HGT-based resistances do not exist in the MTBC (Boritsch *et al*. 2016; Gagneux 2018), competition between clones (i.e. clonal interference) is likely to play an important role in determining the evolutionary fate of emerging AMR mutants. Specifically, the less costly a given AMR mutation, the less likely the AMR mutant would be outcompeted by its wild-type counterpart. This would consequently lead to a greater likelihood of observing heteroresistance prior to antimicrobial exposure. In the MTBC, fitness cost for isoniazid-resistance was first observed in the 1950s, where laboratory-derived and clinical isolates of isoniazid-resistant strains showed lower virulence compared to the isoniazid-susceptible strains in animal models (Barnett, Bushby and Mitchison 1953; Middlebrook and Cohn 1953). Due to the fitness cost that AMR mutations confer, it was originally hypothesized that MDR-TB would remain a local public health problem (Dye *et al*. 2002). While global MDR incidence has indeed remained stable during the past years at approximately 3% of new TB cases and 18% of previously treated cases (WHO 2020), there have been multiple documented cases of community- or country-wide MDR-TB and XDR-TB transmission (de Vos *et al*. 2013; Leung *et al*. 2013; Casali *et al*. 2014; Eldholm *et al*. 2015; Shah *et al*. 2017; Wollenberg *et al*. 2017; Yang *et al*. 2017; Merker *et al*. 2018), with some countries in Eastern Europe and Central Asia having >25% of new TB cases being MDR-TB (WHO 2020). Many of these MDR-TB transmission clusters were associated with low-cost AMR mutations. For instance, the *rpoB* S450L mutation has been shown to confer little or no fitness costs *in vitro* (Gagneux *et al*. 2006; Song *et al*. 2014), and is generally both the most prevalent rifampicin-resistance mutation and the most strongly associated with MDR-TB transmission (Casali *et al*. 2014; Farhat *et al*. 2016; Wollenberg *et al*. 2017; Yang *et al*. 2017; Merker *et al*. 2018). Positive associations between the *in vitro* fitness of AMR mutations and their relative frequency in the clinic have also been observed for streptomycin-resistance (Sander *et al*. 2002; Nhu *et al*. 2012; Jagielski *et al*. 2014) and for fluoroquinolone-resistance (Avalos *et al*. 2015; Castro *et al*. 2020). Thus, in general, while high-cost mutations are the majority of expected evolutionary outcomes from an initially susceptible MTBC population, the emergence of rare low-cost mutations may allow for the maintenance of heteroresistance within a patient, which can lead to the establishment of AMR upon treatment.

Epistasis, defined as the phenomenon where the phenotypic effect of a given mutation is modulated by the presence of another or multiple other mutations, may also modulate the prevalence of heteroresistance and of AMR. One well-studied example of this in the MTBC are compensatory mutations, which are mutations that alone may confer no fitness benefits or even a fitness cost, but when co-occurring with a bona fide AMR mutation alleviates the fitness costs of that AMR mutation (Fig. 2A). Mutations in *rpoA* and *rpoC* have been confirmed to compensate for fitness costs of rifampicin resistance-conferring mutations in *rpoB* (Comas *et al*. 2012; Song *et al*. 2014). Further, these compensatory mutations have been shown to associate with large MDR-TB transmission clusters in South Africa (de Vos *et al*. 2013), Russia (Casali *et al*. 2014), China (Li *et al*. 2016) and Uzbekistan (Merker *et al*. 2018). Notably, compensatory mutations are more frequently observed with the low-cost rifampicin-resistance *rpoB* S450L mutation compared to other, more costly rifampicin-resistance mutations (de Vos *et al*. 2013; Casali *et al*. 2014; Merker *et al*. 2018). While this may seem counterintuitive at first, this may be explained by two potential mechanisms. Firstly, during infection, AMR mutant populations with low-cost mutations are less likely to be driven to extinction due to clonal interference compared to AMR mutants with high-cost mutations. Second, AMR mutant populations with a low-cost mutation would also experience a greater likelihood of producing new genetic variants in a given unit of time due to a higher reproductive rate. Compensatory mutations in *ahpC* for isoniazid-resistance further confirm the importance of these mutations in AMR evolution (Sherman *et al*. 1996). However, unlike compensatory mutations in *rpoA* and *rpoC*, *ahpC* mutations do not appear to associate with transmission. This suggests that while compensatory mutations may improve within-host evolutionary success in general, different compensatory mutations may differently impact the transmissibility of AMR mutants.

Epistasis between different AMR mutations may predispose a population of monoresistant MTBC strains to become resistant to additional drugs. For instance, it has been shown that laboratory-derived isoniazid-resistance MTBC strains were more likely to acquire *rpoB* S450L *in vitro* compared to isogenic drug-susceptible strains (Bergval *et al*. 2012). Similar epistasis between AMR mutations relevant to MTBC have been shown using model organisms, with epistasis between rifampicin-resistance and fluoroquinolone-resistance mutations present in *M. smegmatis* (Borrell *et al*. 2013), and between rifampicin-resistance and streptomycin-resistance present in *E. coli* (Durão *et al*. 2015). However, whether epistasis is a significant modulator in the stepwise acquisition of AMR mutations in clinical populations of MTBC has yet to be determined. Indeed, while laboratory-derived isoniazid-resistant MTBC strains were more likely to acquire *rpoB* S450L *in vitro*, clinically-isolated isoniazid-resistant MTBC strains were not associated with any particular *rpoB* mutation (Bergval *et al*. 2012). As suggested by the authors, this may be due to the fact that *katG* S315T is frequent in the clinic but has yet to be isolated *in vitro*; because *katG* S315T is likely a low-cost mutation (Pym, Saint-Joanis and Cole 2002), there is likely less of a selection pressure for *katG* S315T to acquire low-cost rifampicin-resistance mutations such as *rpoB* S450L. A recent study also suggested that positive epistasis between two normally rare rifampicin- and fluoroquinolone-resistance mutations may have allowed for both to become the predominant mutations in a pre-XDR-TB patient, but laboratory experiments are required to confirm this epistasis (Yoshida *et al*. 2020). Thus, while epistasis between AMR mutations may occur, current transmission of AMR in MTBC appears to be driven more by the individual fitness cost of mutations coupled with compensatory mutations rather than by epistasis between AMR mutations.

Epistasis between AMR mutations and the bacterial genotype may also determine the magnitude of heteroresistance. One previous example that lends support for this type of epistasis has been the association between L2 Beijing strains and MDR-TB (Borrell and Gagneux 2009; Casali *et al*. 2014; Merker *et al*. 2015; Eldholm *et al*. 2016; Wollenberg *et al*. 2017). L2 Beijing strains have been shown to associate with the low-cost isoniazid-resistance *katG* S315T (Fenner *et al*. 2012) and with low-cost rifampicin-resistance *rpoB* S450L mutations (Fenner *et al*. 2012; Casali *et al*. 2014). This suggests that L2 Beijing strains may more readily acquire such low fitness cost AMR mutations. MTBC genotype-dependent AMR mutation fitness effects have also been observed for other anti-tuberculosis drugs, such as fluoroquinolone-resistance mutations having different *in vitro* fitness costs depending on in which MTBC genetic background they were present (Castro *et al*. 2020; Fig. 2B).

The fitness of a given mutation is also dependent on the environment in which it is present. Recent work in mice infected with *E. coli* showed that the microbiome composition present in the host modulated the fitness of common rifampicin- and streptomycin-resistance mutations (Leónidas Cardoso *et al*. 2020). Specifically, changes in the composition of the microbiome may lead to differences in the resources present (Leónidas Cardoso *et al*. 2020); this in turn may lead to changes in the ecological interactions present such as competition and, consequently, the individual fitness of bacterial strains (Leónidas Cardoso *et al*. 2020). Mouse infection models with the malarial parasite *Plasmodium chabaudi* showed similar environment-dependent strain fitness, as limiting the nutrient paraaminobenzoic acid led to a lower competitive ability of mutants resistant to the antimalarial drug pyrimethamine compared to their wild-type counterparts, leading to the prevention of pyrimethamine-resistance emergence (Wale *et al*. 2017). Interestingly, in the MTBC, the most common isoniazid-resistance mutation in the clinic, *katG* S315T (Casali *et al*. 2014; Vilchèze and Jacobs Jr 2014; Seifert *et al*. 2015), confers a low fitness cost in animal models (Pym, Saint-Joanis and Cole 2002). However, *in vitro* isolation of *katG* S315T has proven elusive, and it has been hypothesized that *katG* S315T may be costly *in vitro*, but not *in vivo* (Bergval *et al*. 2009; Brossier *et al*. 2016). A similar scenario has been observed for the fluoroquinolone-resistance mutation *gyrA* D94A, which is the third-most prevalent fluoroquinolone-resistance mutation in the clinic but rare *in vitro* (Avalos *et al*. 2015; Castro *et al*. 2020). The discrepancy between the frequency of *gyrA* D94A mutation *in vitro* versus in the clinic may be because (1) the antibiotic concentration *in vitro* was too high for it to be observed, or (2) its fitness cost *in vivo* was much lower than *in vitro*. Nevertheless, environment-dependent AMR fitness costs may occur in the MTBC. Further studies are required to test for host-dependent AMR fitness costs in the MTBC, and whether this modulates the magnitude of heteroresistance *in vivo*.

### Dynamics of genetic diversity in presence of antimicrobials

Antimicrobials are a strong selective pressure on infecting MTBC populations during patient treatment. At the simplest level, we would expect a strong positive selection for the MTBC subpopulations with AMR mutations, leading to them sweeping to fixation. However, the within-host evolution of AMR in MTBC populations in the presence of antimicrobials has been shown to be far more complex. In this section, we review the studies that attempted to observe within-host MTBC population genetic diversity and dynamics across space and over time, with a particular focus on AMR evolution. In general, these studies used WGS of multiple MTBC samples isolated from the same TB infected individuals, usually using serially sampled sputa (Sun *et al*. 2012; Merker *et al*. 2013; Eldholm *et al*. 2014; Liu *et al*. 2015; Trauner *et al*. 2017; Séraphin *et al*. 2019), multiple same-day sampling of sputa (Pérez-Lago *et al*. 2014; Trauner *et al*. 2017) and occasionally samples from different anatomical sites (Pérez-Lago *et al*. 2014; Lieberman *et al*. 2016). We will first discuss the general observation that within-host AMR evolution usually involves the co-existence of multiple AMR clones, with the eventual fixation of a given AMR clone. We will then discuss the ‘branched evolution’ phenomena that can be observed in within-host MTBC studies, and the roles that purifying selection, spatiality and phenotypic drug tolerance can play in the dynamics of MTBC genetic diversity within patients undergoing treatment.

#### Evolutionary fate of AMR mutations

**Figure 3.**
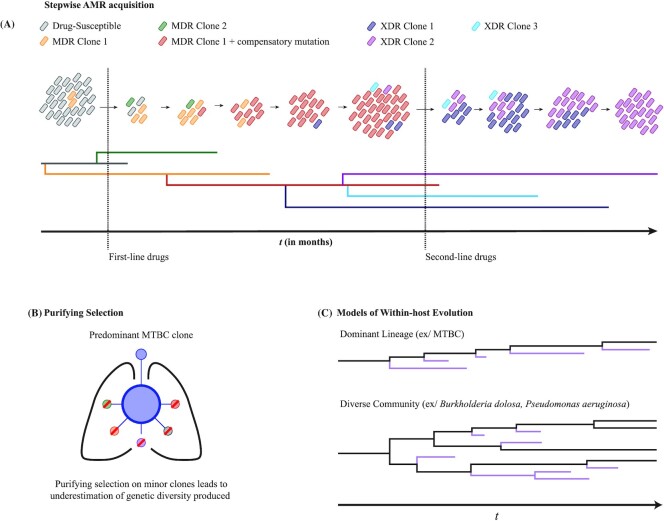
Dynamics of MTBC genetic diversity within-host in the context of AMR. (A) Stepwise acquisition of chromosomal AMR mutations in the MTBC in the presence of antimicrobial pressure. Multiple AMR mutants may emerge from the same parental clonal population, and can co-exist for weeks and even months. Such population dynamics would lead to a ‘branching evolution’ pattern. Compensatory mutations that alleviate AMR mutation costs may also be acquired during the course of infection. However, a single AMR mutant clone appears to outcompete all other AMR mutant clones, and can acquire further mutations to become resistant to subsequently used antimicrobials. Phylogenetic tree used to visualize different bacterial clones over time, with colors denoting their clonality and phenotype (MDR = multidrug-resistant; XDR = extensively drug-resistant). (B) Sufficient antimicrobial pressure appears to confer strong purifying selection pressure on infecting MTBC populations. Purifying selection of minor clones would effectively lead to an underestimation of the MTBC genetic diversity that would have been produced during the course of infection, as the predominant MTBC clone would generally be the only clone sampled from a given patient. (C) Two models have been proposed for how bacterial populations evolve within-host: the Dominant Lineage model and the Diverse Community Model. The MTBC appears to follow the Dominant Lineage model, where new variants may be produced, but co-existence is transient and only one clone dominates the infection long-term. In contrast, the Diverse Community model is characterized by multiple clones maintaining the infection long-term; infections caused by the opportunistic pathogens *Burkholderia dolosa* and *Pseudomonas aeruginosa* appear to follow this model in cystic fibrosis patients (Lieberman *et al*. 2014; Winstanley, O'Brien and Brockhurst 2016; Clark *et al*. 2018). Phylogenetic tree used to visualize different bacterial clones over time; here, black clones are responsible for long-term infections, while purple clones eventually become extinct (Fig. 3B adapted from Trauner *et al*. 2017, with permission).

An initial study using IS*6110* RFLP patterns from sputum isolates from the same TB patient showed that TB patients can be infected with the same MTBC strain for up to 9 years (Mariam *et al*. 2011). However, PCR and Sanger sequencing of AMR genes showed that rather than simple fixation of a given AMR mutant following antimicrobial pressure, both clonal sweeps as well as transient co-existence of different AMR mutant clones occurred during that time (Mariam *et al*. 2011). This suggested extensive dynamics and *de novo* generation of genetic diversity in the infecting MTBC population. More recent studies using WGS have supported this high dynamicity of MTBC genetic diversity within-host. These include studies that used serial MTBC sputum samples collected from patients in China (Sun *et al*. 2012; Trauner *et al*. 2017), in Europe (Merker *et al*. 2013; Eldholm *et al*. 2014) and in the United States (Séraphin *et al*. 2019), showing that these within-host dynamics of MTBC genetic diversity occurs irrespective of the differences in health care systems or human immunity. Such appreciable levels of *de novo* generation of genetic diversity can lead to the phenomenon where heteroresistance can be maintained for long periods, sometimes for years, and include the co-existence of multiple AMR mutants (Mariam *et al*. 2011; Sun *et al*. 2012; Merker *et al*. 2013; Eldholm *et al*. 2014; Trauner *et al*. 2017; Fig. 3A). Even if a given clone was at fixation in a given sputum sample, subsequent samples can show new clones emerging. Sun *et al*. showed that one patient initially had a sputum sample that only contained the rifampicin-resistant clone *rpoB* L533P, but after 18 months, an additional rifampicin-resistant clone with the mutation *rpoB* H526Y was found at a higher frequency than the originally fixed *rpoB* L533P clone (Sun *et al*. 2012). Such a phenomenon may be explained by three possible scenarios. First, the second mutant (in this case, the *rpoB* H526Y mutant) may have spontaneously emerged during the course of treatment and had a lower fitness cost than the first mutant (in this case, *rpoB* L533P). Second, the second mutant was originally present but at a very low frequency and not captured unless deep sequencing was used (Box 1). Third, the second mutant was originally present but in granuloma and cavities that did not provide as much bacteria to the initial sputum sample as the granuloma and cavities containing the first mutant. Indeed, as highlighted in Box 1, sputum sampling biases and the inherent limitations of WGS may lead to variations in detecting the presence of minor genetic variants and in measuring their true proportions. This ultimately affects the interpretation of the within-host evolutionary fate of mutations, such as when mutations emerge and whether they are maintained in the population or driven to extinction.

Irrespective of the roles that either granulomas or low-resolution sequencing plays in observing highly dynamic MTBC genetic diversity, spontaneous emergence of genetic diversity certainly plays a role. This is exemplified by the observation of the stepwise acquisition of AMR mutations to different TB drugs during the course of treatment. Eldholm *et al*. (2014) followed a single patient who was initially infected with a drug-susceptible strain and became XDR-TB through the stepwise acquisition of AMR mutations during treatment. Merker *et al*. (2013) showed similar stepwise acquisition of AMR, with different AMR clones able to compete for long periods until the fixation of a single AMR clone. Clonal interference and complex population dynamics need not be long-lived. Liu *et al*. (2015) showed that three different MDR-TB clones could be detected at changing frequencies over the span of 8 weeks. Similarly, Trauner *et al*. (2017) used deep sequencing (∼1000-fold coverage) and observed two MTBC mutants harboring different fluoroquinolone-resistance mutations emerging at different times, competing, and then one reaching fixation within 8 weeks. Further, deep sequencing showed larger levels of genetic diversity within TB patients than previous studies, where minor alleles made up the majority of the genetic diversity present. This phenomenon was confirmed in recent works by Séraphin *et al*. (2019) and Liu *et al*. (2020b). Thus, while fixation of an AMR clone will eventually occur, there appears to be a large production of MTBC genetic diversity within-host, as well as more dynamicity than a simple clonal sweep of a single AMR clone following onset of treatment.

#### Branched evolution

The continuous production of genetic diversity and highly dynamic nature of MTBC evolution within-host can lead to a ‘branched evolution’ pattern. Branched evolution is characterized by the independent emergence of multiple different subclones from an initially monoclonal population. Branched evolution can be observed when comparing MTBC strains between hosts, such as when a TB patient becomes a ‘super spreader’ and infects multiple secondary hosts (Gardy *et al*. 2011; Walker *et al*. 2013; Pérez-Lago *et al*. 2014; Lee *et al*. 2020). In the context of AMR, branched evolution is clearly observed with the co-existence of different AMR mutants that emerged from the same parental population, as previously highlighted (Merker *et al*. 2013; Eldholm *et al*. 2014; Liu *et al*. 2015; Trauner *et al*. 2017).

Branched evolution may complicate inferring transmission patterns when using genomic sequences. Genetic distances between epidemiologically-linked MTBC strains are generally small (Achtman 2008), so much so that Walker *et al*. (2013) showed that transmission between two MTBC strains may be inferred if they have a genetic distance of only five SNPs or less, while anything above 12 SNPs distance threshold can be considered non-related. Due to the branched evolution within-host, genetic distances between different MTBC genotypes within the same patient may reach or even exceed the five and 12 SNPs distance thresholds (Pérez-Lago *et al*. 2014; Liu et al. 2015, 2020b; Lieberman *et al*. 2016). This can make transmission patterns more difficult to infer. However, recent work also shows that MTBC genetic distances between-host may still be within the five SNPs threshold (Herranz *et al*. 2018; Séraphin *et al*. 2019). Thus, it appears that the genetic variation within-host may not always translate to large genetic distances between-host.

In the context of AMR, mutations in loci that are genetically linked to AMR mutations can concurrently increase in frequency as the AMR mutations themselves are selected for during antimicrobial treatment (i.e. genetic hitchhiking; Maynard Smith and Haigh 1974; Eldholm *et al*. 2014). This scenario can also lead to larger observed genetic distances within-host, again potentially leading to difficulties inferring transmission. Indeed, Walker *et al*. showed that the four out of 30 TB patients they followed longitudinally developed AMR and also showed larger genetic distances (7–11 SNPs) than TB patients that did not develop AMR and were not cases of mixed infections (Walker *et al*. 2013). Using a five SNPs cut-off may miss transmission events of AMR mutants in such a scenario.

#### Purifying selection and background selection

In the cases highlighted above, AMR mutants were fixed in the population due to the strong positive selection imposed by the antimicrobial. However, approximately 85% of all treated drug-susceptible TB cases have positive outcomes (Farah *et al*. 2005; Bao, Du and Lu 2007; Gebrezgabiher *et al*. 2016; Tiberi *et al*. 2018). MTBC population dynamics in the positive treatment outcomes must be inherently different than dynamics where treatment failed due to AMR, as the latter led to the proliferation and fixation of AMR mutants while the former did not. Indeed, Trauner *et al*.(2017) showed that when an effective drug treatment regimen consisting of four or more drugs were present, MTBC population dynamics within-host showed clear signs of purifying selection (Fig. 3B). Independent of the antimicrobials, signatures of purifying selection has been previously demonstrated in between-host (Pepperell *et al*. 2013) and recently in within-host MTBC studies (Liu *et al*. 2020b). This may be because there is likely a limited number of evolutionary trajectories that allow for the MTBC to become more fit due to the MTBC being an obligate and human-adapted pathogen, leading to most new genetic variants emerging within-host being selected against (Brites and Gagneux 2012). This would lead to a stark difference in how the MTBC evolves within-host compared to other non-obligate bacterial pathogens. In a seminal study, Lieberman *et al*. (2014) showed that long-term infections of *Burkholderia dolosa* in cystic fibrosis patients demonstrated a ‘Diverse Community’ model of evolution, whereby an initial infecting bacterial lineage gave rise to multiple different bacterial lineages that were maintained at appreciable population levels throughout the course of infection (Nguyen and Singh 2006). During infections caused by *P. aeruginosa* in cystic fibrosis patients, multiple *P. aeruginosa* lineages also appeared to maintain long-term infections (Marvig *et al*. 2013; Markussen *et al*. 2014; Jorth *et al*. 2015; Winstanley, O'Brien and Brockhurst 2016; Clark, Guttman and Hwang 2018). In contrast, the MTBC appears to follow a ‘Dominant Lineage’ model of evolution within mice (Copin *et al*. 2016) and in humans (Trauner *et al*. 2017), whereby the initial infecting bacterial lineage can give rise to multiple new genetic variants, but most new genetic variants are generally lost or found at very low frequencies, and a single dominant lineage maintains long-term infection instead (Fig. 3C). This is a hallmark of purifying selection, and when combined to the action of background selection (whereby mutations linked to a deleterious mutation are also lost due to lack of recombination), likely leads to a reduction in the within-host MTBC genetic diversity that can be observed. Within a human host and in the presence of antimicrobials, the presence of multiple effective drugs likely further constrains the limited number of evolutionary trajectories available (Trauner *et al*. 2017). By contrast, in the context of ineffective treatment, there appeared to be a relaxation on the restriction of evolutionary trajectories, leading to the rise of AMR mutants and treatment failure (Trauner *et al*. 2017).

Irrespective of AMR, the constant production of MTBC genetic diversity during infection and strong purifying selection may influence other aspects of how the MTBC evolves within-host. For instance, constant genetic diversity production within-host may modulate the virulence (i.e. the pathogen-induced reduction of host fitness) and transmissibility of MTBC strains following chronic infection (Box 2).

Box 2. **Short-sighted evolution in the MTBC?**

Recent studies have shown new MTBC genetic variants appearing independently of AMR in both animal models (Copin *et al*. 2016) and TB patients (Séraphin *et al*. 2019). However, little is known on the consequences of genetic diversity generation on virulence evolution and its implications for transmissibility in the MTBC. It can be hypothesized that appreciable levels of genetic diversity emerging within patients may provide opportunities for MTBC populations to acquire adaptations to their immediate host environment in order to better extract resources from their hosts or modulate host immunity to the pathogen's advantage, effectively increasing virulence. With respect to the host immune pressure, increasing evidence has shown that most human T cell epitopes in MTBC are evolutionarily hyperconserved (Comas *et al*. 2010; Pepperell *et al*. 2013; Coscolla *et al*. 2015; Stucki *et al*. 2016). While the underlying reason for this observation needs further study, some T cell epitopes in clinical strains are diverse (Coscolla *et al*. 2015), and recent data from mouse models indicate that T cells drive diversification of certain epitopes in MTBC (Copin *et al*. 2016). In addition, the PE/PPE/PGRS families of genes known to be highly variable have been hypothesized to be involved in virulence and antibody escape (Copin *et al*. 2014; Singh *et al*. 2016; Wang *et al*. 2020). Recent work further reported signals of positive selection in MTBC genes that are linked to host survival and immune response modulation (Vargas *et al*. 2020). However, increased adaptation to their immediate host environment may come at the cost in their ability to transmit to the next host, a phenomenon referred to as ‘short-sighted evolution’ (Levin and Bull 1994), so much so that the ancestral and less virulent strain is generally the strain that successfully transmits to the next host. In HIV, short-sighted evolution has been invoked to explain the observation that viral virulence increases during the course of infection in an individual patient, but most of the transmission to new patients involves ‘early’ (i.e. ancestral) viral variants (Troyer *et al*. 2005; Kouyos *et al*. 2011; Lythgoe *et al*. 2017). In the MTBC, the most extreme example of this is TB meningitis and other extrapulmonary cases of TB, where the extrapulmonary bacilli cannot transmit and therefore represent ‘evolutionary dead-ends’ (Gagneux 2018). Indeed, MTBC has to cause pulmonary disease to transmit, thus leading to a trade-off between virulence and transmission (Gagneux 2018). This notion is supported indirectly by epidemiological data indicating that before the wide-spread availability of anti-retroviral treatment, TB patients co-infected with HIV were less likely to transmit TB because (i) HIV/TB patients are more likely to have extrapulmonary (i.e. non-transmissible) TB and (ii) because these patients die faster than HIV-uninfected TB patients (Brites and Gagneux 2012). Thus, similar to HIV (Lythgoe *et al*. 2017), given the close association of MTBC with its human host (Brites and Gagneux 2015), bacterial populations within a patient might adapt to this particular immunological environment, which might come at the cost of a reduced capacity to establish a new infection in a secondary host—a notion that has, however, never been tested.

The previously underappreciated MTBC genetic diversity dynamics present during infection within-host begs the question: how does the MTBC generate such apparently extensive genetic diversity when previous studies inferred low mutation rates? Recent work by Morales-Arce *et al*. (2020) suggest that MTBC clonality, the resulting skewed progeny distribution and purifying selection (i.e. forces that all reduce the observed genetic diversity present) result in an underestimation of MTBC mutation rates within-host when using evolutionary frameworks centered around the Wright–Fisher model. Use of evolutionary models that take clonality and skewed progeny distributions into account, such as multiple-merger coalescent theory (Menardo, Gagneux and Freund 2020; Morales‐Arce *et al*. 2020), may allow for a better estimate of MTBC genetic diversity production, including testing whether different MTBC genetic backgrounds indeed have different mutation rates.

#### Role of spatiality: between-lesion bacterial migration and clonal interference

**Figure 4.**
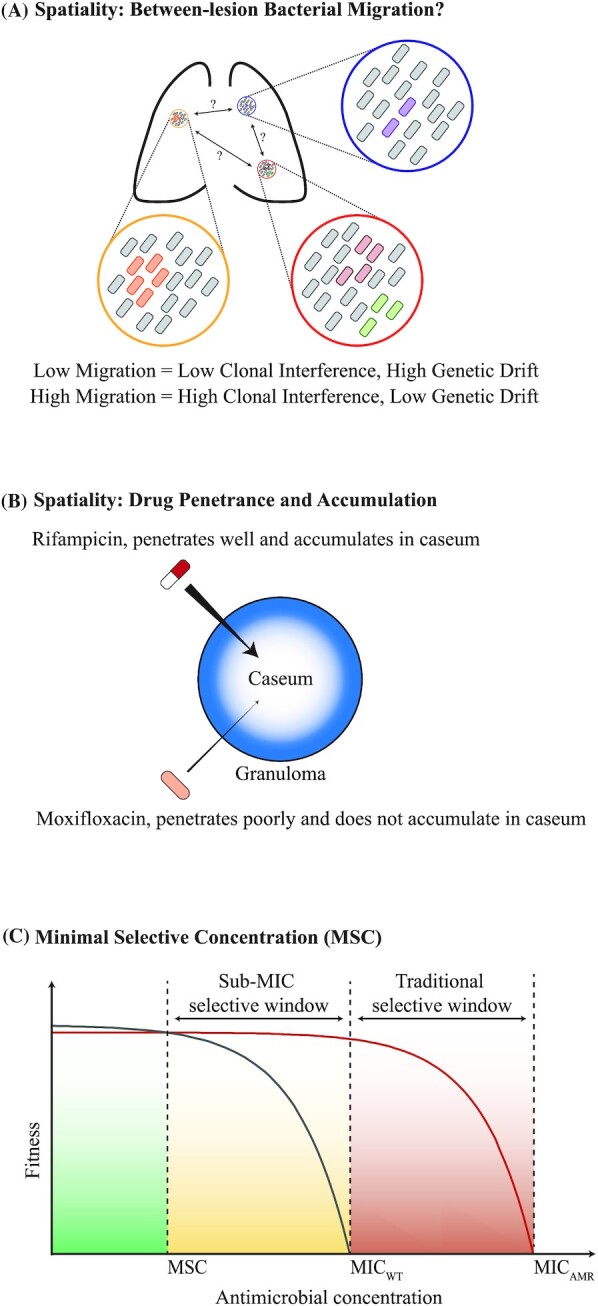
Role of spatiality in the within-host evolution of AMR in the MTBC. (A) MTBC populations may be spatially-segregated in different lung lesions. Not much is known about population mixing between these different lesions. Hypothetically, low levels of migration between lesions would lead to lower clonal interference, a greater role of genetic drift and higher regional genetic differentiation. In contrast, high migration levels would lead to greater clonal interference, smaller role of genetic drift and less regional differentiation. (B) Different anti-TB drugs have been shown to have different capacities to penetrate into granulomas. This leads to differences in antimicrobial accumulation rates and total concentrations in the caseum, the lesion site that generally harbors the greatest density of MTBC bacilli. General pharmacokinetics of rifampicin and moxifloxacin are shown here as an example. (C) Concept of minimal selective concentration (MSC). Antimicrobials impose a dose-dependent reduction in the fitness of bacteria. Although the fitness of drug-susceptible wild-type and AMR mutants is zero at their respective minimum inhibitory concentrations (MIC_WT_ and MIC_AMR_ = minimum inhibitory concentration for wild-type and AMR mutant, respectively), the fitness reduction occurs earlier in the antimicrobial concentration gradient for wild-type compared to the AMR mutant. The antimicrobial concentration where the fitness of the wild-type is equivalent to the fitness of the AMR mutant is the MSC. The MSC can be well below MIC_WT_, and antimicrobial concentrations above this point will already select for the AMR mutant (Fig. 4C adapted from Gullberg *et al*. 2011, with permission).

Pathophysiological substructures in the lungs of TB patients may modulate the dynamics of within-host MTBC genetic diversity. Multiple lesions are likely present in any given TB patient (Cadena, Fortune and Flynn 2017; Strydom *et al*. 2019), with each lesion possibly harboring different MTBC genotypes (Lin *et al*. 2014; Liu *et al*. 2015; Dheda *et al*. 2018). Consequently, MTBC populations in different tissue sites within the same patient may have larger genetic distances than what is observed between two MTBC strains from different patients (Pérez-Lago *et al*. 2014; Lieberman *et al*. 2016). In autopsy samples from HIV co-infected TB patients who died prior to treatment, Lieberman *et al*. showed that MTBC genetic distances did not correlate with the proximity of the tissue in which the different MTBC variants were found (Lieberman *et al*. 2016). Similar work using a macaque infection model by Lin *et al*. showed that each lesion could have different and non-overlapping evolutionary trajectories (Lin *et al*. 2014). Furthermore, each lesion appeared to be founded by a single MTBC genotype, and each lesion may respond differently to treatment (Lin *et al*. 2014; Liu *et al*. 2015). However, not much is known about the extent of MTBC population movement between different lesions (Fig. 4A). Such regional isolation of bacterial subpopulations have also been observed in *P. aeruginosa* infections in cystic fibrosis patients (Markussen *et al*. 2014; Jorth *et al*. 2015; Winstanley, O'Brien and Brockhurst 2016; Clark, Guttman and Hwang 2018). Moreover, there appeared to be limited bacterial subpopulation mixing between the different *P. aeruginosa* infection sites, leading to divergent evolution (Markussen *et al*. 2014; Jorth *et al*. 2015; Winstanley, O'Brien and Brockhurst 2016; Clark, Guttman and Hwang 2018). Hypothetically, lower levels of population movement between lesions should lead to greater branching evolution, as establishment of the MTBC or *P. aeruginosa* populations in each lesion would be predisposed to a ‘founder effect’ (Lin *et al*. 2014; Markussen *et al*. 2014; Jorth *et al*. 2015). There would also be an increased role for genetic drift in determining the genetic diversity in the patient, as each lesion would constitute a distinct population with a smaller effective population size (Ellegren and Galtier 2016).

Spatial substructuring likely modulates clonal interference dynamics as well (Lin *et al*. 2014; Markussen *et al*. 2014; Jorth *et al*. 2015). Specifically, the lower the bacterial migration levels are between different lesions, the less of an effect clonal interference will have on the within-host evolutionary dynamics of AMR in the MTBC. This is because low migration levels effectively lead to distinct MTBC subpopulations in one lesion experiencing reduced competition by MTBC subpopulations in other lesions. Recent *in vitro* work in *E. coli* shows that reduced competition due to spatial segregation may allow for the maintenance of multiple AMR mutations for longer durations compared to populations without spatial segregation (Durão *et al*. 2020). Thus, for the MTBC, low between-lesion migration levels would hypothetically lead to higher levels of MTBC genetic diversity and, consequently, higher likelihood of heteroresistance observed within TB patients. However, the extent of MTBC between-lesion migrations in the lungs of patients has yet to be determined.

#### Role of spatiality: pharmacokinetics and drug penetrance

The duration and extent that antimicrobials are present in the MTBC infection sites likely modulates within-host bacterial population dynamics. Indeed, the heterogeneity in the spatial and temporal availability of antimicrobials determines both the strength of the selection pressure for AMR mutants in a heteroresistant population, as well as the effective bacterial population size (which, as previously discussed, can modulate the magnitude of heteroresistance). This spatiotemporal heterogeneity in antimicrobial concentration can be influenced by multiple factors. Firstly, the nature of TB lesions may differ between different TB patients, and even within a given patient, leading to differences in antimicrobial concentrations (Dartois 2014; Liu *et al*. 2015; Cadena, Fortune and Flynn 2017; Dheda *et al*. 2018; Strydom *et al*. 2019). Granulomas exhibit a spectrum of possible structures, with each type having different immunological properties in controlling the TB infection (reviewed in Cadena, Fortune and Flynn 2017 and Pagán and Ramakrishnan 2018). Different granuloma types can be found simultaneously within the same patient (Lin *et al*. 2014; Prideaux *et al*. 2015; Dheda *et al*. 2018; Strydom *et al*. 2019; Cicchese *et al*. 2020). In general, granulomas first form as cellular granulomas, where macrophages, lymphocytes and blood cells intertwine inside a shell of fibroblasts. In this form, MTBC bacilli are present and actively growing intracellularly in macrophages and extracellularly, and antimicrobials can easily penetrate the center of the granuloma due to ample blood supply (Dartois 2014). Granulomas can continue to grow and mature into one of multiple types of granulomas (Cadena, Fortune and Flynn 2017; Pagán and Ramakrishnan 2018). Their general structure can be characterized by a necrotic and acellular center called the caseum, which lacks blood vessels and is surrounded by densely packed macrophages, lymphocytes and occasionally fibroblasts. In mature granulomas, bacteria may reside intracellularly in macrophages and extracellularly in the caseum (Dartois 2014), but the extracellular subpopulation are often in a non-replicating metabolic state (the likely consequences of which will be discussed in the next subsection; Sarathy *et al*. 2018). If a granuloma comes into contact with an airway, the caseum can fuse with the airway, leading to a cavitary lesion (Dartois 2014). Such cavitary lesions are generally associated with failed treatment, higher transmission rates and AMR (Cegielski *et al*. 2014; Mbuagbaw *et al*. 2019; Urbanowski *et al*. 2020). The lack of vascularization in the caseum, in particular, imposes a challenge for antimicrobial availability. Specifically, an antimicrobial gradient forms where the caseum and the cavitary-caseum interface contain the lowest antimicrobial concentrations compared to other lesion sites (Pienaar *et al*. 2015; Prideaux *et al*. 2015; Blanc *et al*. 2018; Dheda *et al*. 2018; Sarathy *et al*. 2018; Strydom *et al*. 2019; Ordonez *et al*. 2020). As the caseum usually harbors the largest bacterial burden, the majority of the bacilli population are therefore likely not exposed to sterilizing concentrations of antimicrobials (Pienaar *et al*. 2015; Ordonez *et al*. 2020). Recent simulations using relevant antimicrobial pharmacokinetic parameters in TB patients also suggest that larger granulomas are likely to experience less antimicrobial concentrations at their center (Cicchese *et al*. 2020). This is particularly important, as lesions can be up to 1000 cm^3^ in volume (Dheda *et al*. 2018). Taken together, granuloma heterogeneity can lead to spatiotemporal heterogeneity in antimicrobial concentrations, with the caseum generally having the lowest antimicrobial concentrations. Furthermore, granuloma heterogeneity may explain lesion-specific sterilization patterns observed in TB patients (Lin *et al*. 2014; Liu *et al*. 2015; Dheda *et al*. 2018; Strydom *et al*. 2019; Ordonez *et al*. 2020), which in turn leads to variable selection pressures on MTBC populations.

Independent of granuloma heterogeneity, different TB drugs also differ in their capacity to penetrate host tissue. Important work by Prideaux *et al*. (2015) showed heterogeneity in the lesion penetrance for four anti-tubercular drugs (isoniazid, rifampicin, pyrazinamide and the fluoroquinolone moxifloxacin) in the lungs of TB patients. For example, rifampicin accumulated in the caseum while moxifloxacin did not (Prideaux *et al*. 2015; Fig. 4B). Recent studies further confirm drug-specific lesion penetrance in TB patients (Kempker *et al*. 2017; Dheda *et al*. 2018; Heinrichs *et al*. 2018; Strydom *et al*. 2019; Ordonez *et al*. 2020), including between drugs belonging to the same drug class like in fluoroquinolones (Pienaar *et al*. 2017; Sarathy *et al*. 2019). Different drugs also have different half-lives (i.e. the estimated period of time for a given drug to be reduced to half its concentration in the body), which further contributes to the spatiotemporal variability in the concentration of different drugs (McIlleron *et al*. 2006; Tostmann *et al*. 2013; Wilby and Hussain 2020). Host genetics, the age of the patients, the dosing of the drug, drug–drug interactions, food–drug interactions and co-morbidities such as HIV and diabetes further modulate the levels of antimicrobials present (McIlleron et al. 2006, 2015; Tostmann *et al*. 2013; Abulfathi *et al*. 2019; Erwin *et al*. 2019; McIlleron and Chirehwa 2019; Huynh *et al*. 2020). Such drug-specific heterogeneity, coupled with the fact that different drugs have different modes of action (reviewed in Gygli *et al*. 2017 and Cohen *et al*. 2019), further contribute to the variable drug pressure on different MTBC subpopulations within-host (Pienaar *et al*. 2015; Cicchese *et al*. 2020).

The literature above suggests that spatiotemporal heterogeneity in antimicrobial concentrations can lead to two potential mechanisms that can potentiate the emergence of multidrug-resistance in MTBC. Firstly, Prideaux *et al*. (2015) and Strydom *et al*. (2019) showed that antimicrobial-dependent tissue penetrance can lead to some lesion sites effectively experiencing monotherapy, meaning only one drug is present at concentrations that would sterilize the wild-type strain. Spatial monotherapy may promote multidrug-resistance acquisition as bacterial populations can acquire AMR mutations in a stepwise manner (Moreno-Gamez *et al*. 2015; Strydom *et al*. 2019). However, Dheda *et al*. (2018)recently showed that in MDR-TB patients who failed second-line treatment, only a minority of lesion sites experienced spatial monotherapy, suggesting that increased MTBC resistance levels in other lesion sites (as measured by minimum inhibitory concentration, or MICs) were due to mechanisms independent of spatial monotherapy. Indeed, Dheda *et al*. (2018)found that increased MICs in lesion-specific MTBC subpopulations were associated with low antimicrobial concentrations per lesion site in general (i.e. multiple antimicrobials were present, but at low concentrations). Therefore, Dheda *et al*. (2018)suggested that lower antimicrobial concentrations (as opposed to monotherapy *per se*) were more important at least for how MTBC evolves from MDR-TB to become XDR-TB. Studies in other bacterial pathogens support Dheda *et al*.’s (2018) conclusions (Gullberg *et al*. 2011; Liu *et al*. 2011; Greenfield *et al*. 2018). From an evolutionary standpoint, antimicrobials need not be at concentrations above the MIC of wild-type strains (MIC_WT_) in order to select for AMR mutants. Works by Gullberg *et al*. (2011) and Liu *et al*. (2011)showed that concentrations well below the MIC_WT_ for *E. coli* and *Salmonella* strains led to a dose-dependent reduction on strain fitness, with the fitness reaching zero at MIC_WT_ (Gullberg *et al*. 2011; Liu *et al*. 2011; Fig. 4C). In contrast, dose-dependent reduction on the fitness of isogenic AMR mutants only began at much higher antimicrobial concentrations, which were usually higher than MIC_WT_ (Gullberg *et al*. 2011; Greenfield *et al*. 2018). This effectively leads to a sub-MIC_WT_ antimicrobial concentration where the fitness of the wild-type is already equivalent to the fitness of the AMR mutant (termed as the ‘minimal selective concentration,’ or MSC). The MSC can be more than one order of magnitude lower than the MIC_WT_, and any antimicrobial concentration above MSC would already select for AMR mutants (Gullberg *et al*. 2011; Liu *et al*. 2011; Greenfield *et al*. 2018). Thus, current experimental evidence both in the MTBC and in other bacterial pathogens gives support for the roles of low antimicrobial concentrations and spatial monotherapy in AMR evolution in MTBC. Which of these is more important in driving AMR emergence requires further investigation. Regardless, AMR emergence in the MTBC may be best suppressed by treatment regimens that promote better lesion penetrance of antimicrobials in combination.

#### Role of bacterial phenotypic drug tolerance and persisters

In many bacteria, exposure to sub-inhibitory concentrations of individual antibiotics leads to enhanced phenotypic drug tolerance (Dörr, Vulić and Lewis 2010; Van den Bergh, Fauvart and Michiels 2017). Tolerance refers to the phenotype where drug-susceptible bacteria exhibit prolonged survival when exposed to concentrations of bactericidal antibiotics above MIC_WT_ (Balaban *et al*. 2019; Bakkeren, Diard and Hardt 2020). Cells exhibit tolerance because they are in a non-replicating and/or metabolically quiescent state, and are referred to as ‘persisters’ (Balaban *et al*. 2019; Bakkeren, Diard and Hardt 2020). Tolerance therefore differs from AMR as tolerance is characterized by survival but lack of replication during bactericidal antimicrobial exposures, whereas AMR mutants have active replication and experience population growth (Balaban *et al*. 2019; Bakkeren, Diard and Hardt 2020). Moreover, persisters remain genetically susceptible to the antimicrobial if they are allowed to regrow and are then re-exposed to the same antimicrobial (i.e. their MICs for the antimicrobial does not change; Balaban *et al*. 2019; Bakkeren, Diard and Hardt 2020). Persistence is the observation where only a subpopulation exhibit the tolerance phenotype, and may therefore be referred to as ‘heterotolerance’ (Balaban *et al*. 2019; Bakkeren, Diard and Hardt 2020).

**Figure 5.**
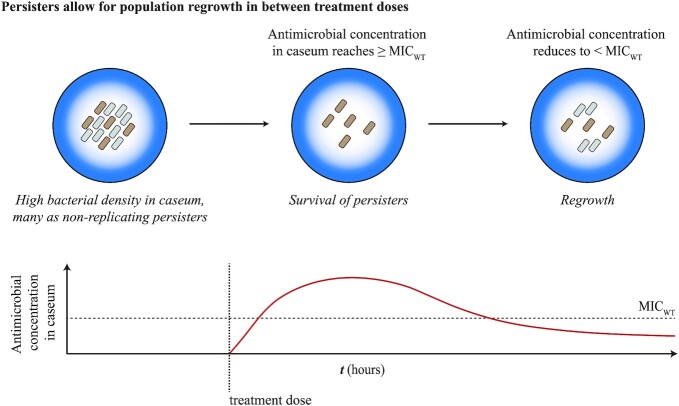
Tolerance and persisters. Tolerance is defined as the ability of drug-susceptible bacteria to survive for an extended period antimicrobial concentrations that would normally kill them (i.e. MIC_WT_). Tolerance is hypothesized to be responsible for the long treatment regimens required to achieve sterility in MTBC infections. Persistence may be defined as ‘heterotolerance,’ when only a subset of the population exhibits the tolerance phenotype. Bacterial cells that exhibit the tolerance phenotype are known as ‘persisters,’ and the granuloma caseum likely harbors large populations of non-replicating MTBC persisters. In between treatment doses, antimicrobial concentrations may drop below MIC_WT_. Persisters may revert back to a replicating phenotype during this period and lead to population regrowth.

Recent work in *Salmonella*, *Mycobacterium marinum* and the MTBC show that persister formation may occur from diverse types of environmental stresses independent of antimicrobial pressure, including induction by host immune pressure during intracellular growth in macrophages, growth in granuloma models of infection, and in mice (Adams *et al*. 2011; Kapoor *et al*. 2013; Helaine *et al*. 2014; Manina, Dhar and McKinney 2015; Liu *et al*. 2016). Persister formation may also occur from errors in the biochemical mechanisms of the bacteria, such errors in translation leading to increased tolerance to rifampicin (Javid *et al*. 2014). As highlighted in the previous section, extracellular MTBC in the caseum are generally in such a non-replicative state, and they have also been shown to exhibit drug tolerance (Sarathy *et al*. 2018). Persisters are often multidrug-tolerant, i.e. they exhibit prolonged survival in presence of drugs to which they have not previously been exposed (Balaban *et al*. 2019; Bakkeren, Diard and Hardt 2020). Hence, persister formation has been proposed to be one of the reasons for the long treatment required to cure chronic bacterial infections such as TB, as persisters may allow for the population regrowth in between treatment doses (Dhar, McKinney and Manina 2016; Van den Bergh, Fauvart and Michiels 2017; Bakkeren, Diard and Hardt 2020; Fig. 5). Studies in the 1950s already showed that mice infected with MTBC could not be sterilized despite extended drug treatment (McCune and Tompsett 1956). Moreover, it is well documented that between 5–10% of TB patients relapse after successfully completing their treatment course, even under ideal clinical trial conditions, and those patients who relapse usually harbor drug-susceptible bacteria (Mirsaeidi and Sadikot 2018; Tiberi *et al*. 2018). The clinical relevance of tolerance is further supported by the observation that in *P. aeruginosa*, *Candida albicans* and *Staphylococcus epidermis*, so-called high-persister mutants (i.e. mutants that exhibit higher magnitudes of persistence or ‘heterotolerance’) increased over time during patient treatment (LaFleur, Qi and Lewis 2010; Mulcahy *et al*. 2010; Haunreiter *et al*. 2019). Such high-persister mutants have also been observed in *E. coli* isolates from urinary tract infections (Schumacher *et al*. 2015). One study in the MTBC showed that like in previous work in *E. coli* (Fridman *et al*. 2014), high-persister mutants can be selected for *in vitro* by exposure to periodic pulses of high antibiotics concentrations followed by growth without antibiotics (Torrey *et al*. 2016). This study also found that MTBC clinical strains differed in their baseline level of persisters by up to 10 000-fold.

Drug tolerance and persister formation have repeatedly been hypothesized to facilitate the development of AMR in the clinic, with high-persistence mutations that increase the magnitude of persistence likely playing a key role (Dhar, McKinney and Manina 2016; Van den Bergh, Fauvart and Michiels 2017; Bakkeren, Diard and Hardt 2020; Liu *et al*. 2020a). Based on the observation that mutational target sizes were much larger for persistence than for AMR (Amini *et al*. 2011; Girgis, Harris and Tavazoie 2012), high-persister mutations may serve as a ‘stepping-stone’ and potentiate the acquisition of bona fide AMR mutations, leading to higher likelihoods of heteroresistance and AMR (Levin-Reisman *et al*. 2017; Liu *et al*. 2020a). However, only few studies currently support that this actually occurs in the clinic. One *in vitro* study in *E. coli* found that tolerance usually preceded resistance to ampicillin (Levin-Reisman *et al*. 2017). More recent work from two patients infected with *Staphylococcus aureus* causing bacteremia showed that tolerance mutations for vancomycin likely preceded and promoted the acquisition of rifampicin-resistance mutations (Liu *et al*. 2020a). However, there are relatively few studies that have tested for this phenomenon in clinical MTBC infections. One recent study in the MTBC identified mutations in the transcription factor *prpR*, which confers multi-drug tolerance under certain (but not all) growth conditions, including growth in macrophages (Hicks *et al*. 2018). These *prpR* mutations were associated with isoniazid-resistance, but also occurred at low frequencies in drug-susceptible strains, indicating possible stepping-stone tolerance mutations that facilitate AMR emergence in the MTBC.

Recent works also suggest that frameshift mutations in *glpK*, which encodes a glycerol-3-kinase and is necessary for glycerol metabolism, may act as stepping-stone tolerance mutations as well (Bellerose *et al*. 2019; Safi *et al*. 2019). These mutations occur in a homopolymeric region of *glpK*, and led to slower growth and reduced susceptibility to isoniazid, rifampicin and moxifloxacin by inducing a drug-tolerant phenotype (Bellerose *et al*. 2019; Safi *et al*. 2019). These frameshift mutations were transient, with its emergence and loss occurring frequently, leading to a genetic-based and reversible ‘on/off’ switch for the tolerance phenotype (Bellerose *et al*. 2019; Safi *et al*. 2019). Such *glpK* mutations were associated with AMR in the MTBC in the clinic, and emerged independently multiple times (Bellerose *et al*. 2019). Further efforts to study the evolution of *glpK* mutations likely require direct sputum sequencing of clinical MTBC isolates, as regrowing clinical isolates in glycerol-containing media represent a potential bias (Safi *et al*. 2020; Vargas and Farhat 2020; Box 1). Further studies are also required to investigate whether similar transient mutations present in other carbon metabolism genes can potentiate AMR due to temporary increases of drug tolerance. Such mechanisms may play a role in recent observations where bacterial population sizes increased for months in the presence of treatment pressure prior to any known AMR mutations being observed in the population (Ngabonziza *et al*. 2020b).

A recent THP-1 macrophage infection study by Adams *et al*. (2019) demonstrated that macrophages induced increased persistence (i.e. higher levels of heterotolerance) to isoniazid in MTBC strains belonging to L1, L2, L3 and L4. Moreover, Adams *et al*. (2019) showed that macrophages also induced increased persistence to rifampicin in L1, L3 and L4 strains, but not in L2 Beijing strains. This study therefore suggests that the magnitude of persistence or heterotolerance is dependent on the bacterial genotype. However, caution must be taken regarding the authors’ conclusion that L2 Beijing strains may not exhibit macrophage-induced persistence to rifampicin. Heterogeneity in the environmental stresses may induce differences in the magnitude of persistence induced (Balaban *et al*. 2019; Bakkeren, Diard and Hardt 2020). THP-1 is a human leukemia monocyte-like cell line, and therefore likely exhibits phenotypic differences compared to the circulating human peripheral blood mononuclear cells for which THP-1 cells are used as a surrogate for *in vitro* studies (Riddy *et al*. 2018; Tedesco *et al*. 2018). These phenotypic differences may include differences in increasing persistence in the MTBC. Thus, more studies using different infection and *in vivo* models are required to test whether L2 Beijing strains indeed do not exhibit macrophage-induced persistence. Indeed, whether different MTBC genetic backgrounds have different baseline levels of persistence in general remains to be determined. Further, it is unclear whether prolonged exposure in macrophages *in vivo* would select for high-persistence mutations in the MTBC, and whether this would lead to higher likelihoods of heteroresistance.

One way by which persisters in MTBC might survive antimicrobial exposure is through enhanced drug efflux, a mechanism that appears to be particularly relevant in mycobacteria growing intracellularly (Adams *et al*. 2011; Adams, Szumowski and Ramakrishnan 2014). For instance, recent work by Adams *et al*. (2011) showed that L2 Beijing strains exhibited higher levels of intra-macrophage growth than non-Beijing MTBC strains, and that this increased intra-macrophage growth was abrogated when the L2 Beijing strains were treated with bacterial efflux inhibitor verapamil (Adams *et al*. 2019). However, in contrast to many other bacteria where the role of efflux pumps in clinical AMR is well supported (Du *et al*. 2018), the situation is much less clear for the MTBC (Black *et al*. 2014; Gygli *et al*. 2017). For example, a recent study found that verapamil affects growth not by reducing drug efflux but by a direct effect on the MTBC's membrane potential (Chen *et al*. 2018). Therefore, more work is required to test the role of efflux pumps in both intracellular growth and tolerance phenotypes in the MTBC.

**Figure 6.**
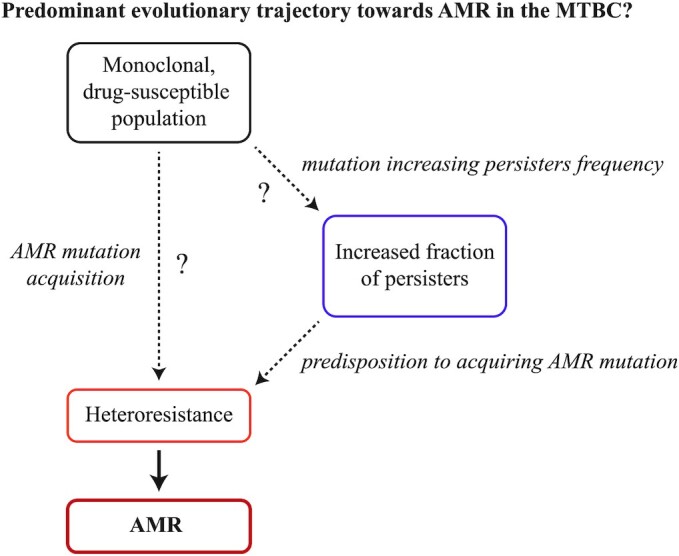
Evolutionary trajectory towards AMR in MTBC. Recent work in other bacteria suggest that mutations that increase the fractions of persisters in the population predispose initially drug-susceptible populations to becoming heteroresistant, and then AMR (Bakkeren *et al*. 2020; Liu *et al*. 2020a). Meanwhile, appreciable levels of genetic diversity production has increasingly been shown to occur during the within-host evolution of the MTBC, and likely play a role in the within-host stepwise acquisition of AMR in MTBC observed during treatment. Considering heteroresistance has been documented for essentially all important anti-TB drug, heteroresistance from the spontaneous emergence of AMR mutations is likely an important factor in AMR evolution in the MTBC. However, whether heteroresistance alone or a ‘persisters-mutations-first’ route is the primary evolutionary trajectory towards AMR in the MTBC, requires further investigation.

Thus, whether high-persistence mutations that increase the fraction of persisters in a population serve as a stepping-stone to becoming heteroresistant, and finally AMR, in the MTBC requires further investigation. Even if high-persistence mutations do serve as a stepping-stone to AMR, it is unclear whether a ‘persistence-mutations-first’ route or heteroresistance alone serves as the major evolutionary trajectory towards AMR in the MTBC (Fig. 6). Indeed, based on recent modeling work showing that rates of genetic diversity production in MTBC may be higher than originally thought (Morales‐Arce *et al*. 2020), acquiring rare, bona fide AMR mutations in MTBC infections may not be so unlikely. More work is therefore required to test the relative contributions of persistence and heteroresistance in the evolution of AMR in the MTBC within-host.

In summary, the previous subsections highlighted the complexities of the within-host dynamics of MTBC genetic diversity. Positive selection, genetic hitchhiking, clonal interference, genetic drift, purifying selection and background selection can all occur in context of AMR evolution within-host. The presence and relative strength of each of these evolutionary forces are likely determined by the heterogeneity in environmental pressures, such as the spatiotemporal heterogeneity of antimicrobials present in different lesion sites within individual patients, as well as differences in bacterial susceptibility to the bactericidal effect of antimicrobials.

## CONCLUSIONS

Recent genome sequencing and experimental work have provided a deeper understanding of how AMR evolves in populations of the MTBC within-host. Biological factors that modulate the generation and maintenance of AMR mutations in bacteria in general, including differential mutation rates, differential population sizes, the breadth of AMR mutations available, and the fitness of AMR mutations, likely determine how AMR evolves in the MTBC. Parallels can be seen in tumor evolution, where differential rates of genetic diversity production, differential population sizes and epistasis can modulate the evolutionary trajectory of tumors (McGranahan and Swanton 2017; van de Haar *et al*. 2019; Vasan, Baselga and Hyman 2019). However, in the MTBC, more work is required to delineate the relative contribution of each of these factors in determining the magnitude of heteroresistance and, consequently, the prevalence of AMR.

What is clear, however, is that compared to opportunistic bacterial pathogens such as *B. dolosa* and *P. aeruginosa*, the MTBC exhibits extreme clonality and a Dominant Lineage model of evolution throughout the course of infection. This clonality stems from two factors. Firstly, the MTBC undergoes no horizontal gene exchange, so new clones almost always compete with existing clones. Secondly, because the MTBC is already well adapted to its human host, new genetic variants in MTBC populations likely experience strong purifying selection. Thus, new genetic variants are less likely to be maintained in MTBC populations within-host. However, recent work also shows appreciable levels of MTBC genetic diversity production within-host. Continuous generation of new genetic variants coupled with strong purifying selection is expected to lead to a significant population turnover. In the context of AMR, this likely leads to the paradoxical situation where MTBC populations appear clonal within-host, but AMR mutations emerge often enough that a combination therapy is required to prevent treatment failure in the clinic. Even under combination therapy, MTBC populations can exhibit a stepwise acquisition of the required AMR mutations to render the combination therapy ultimately ineffective.

Spatiotemporal heterogeneity in pathophysiological structures and antimicrobial concentrations, as well as bacterial phenotypic heterogeneity, also modulate the dynamics of within-host MTBC populations dynamics as well. For instance, spatial segregation likely determines the magnitude of clonal interference within-host. These general characteristics make the within-host evolution of the MTBC similar to what has been observed in *P. aeruginosa* infections in patients with cystic fibrosis (Winstanley, O'Brien and Brockhurst 2016; Clark, Guttman and Hwang 2018). Spatiotemporal heterogeneity in the microenvironment immediately surrounding tumor cells also modulate the evolutionary trajectories of tumors (McGranahan and Swanton 2017; van de Haar *et al*. 2019; Vasan, Baselga and Hyman 2019). However, for the MTBC, the population dynamics within- and between-different lesions, and how these dynamics impact AMR evolution specifically, is still not well understood.

Thus, while the studies we reviewed here have greatly improved our understanding of how the human-adapted MTBC evolves within-host, many open questions remain. Further technological and methodological advances in genome sequencing and patient sampling are vital in future efforts to improve our understanding of the within-host evolution in the MTBC. Such efforts are required to inform the design of more effective treatment strategies, reduce the likelihood of AMR and ultimately prevent further TB transmission.
